# Iron Replacement Attenuates Hypoxic Pulmonary Hypertension by Remodeling Energy Metabolism via Regulating the HIF2α/Mitochondrial Complex I, III/ROS Axis

**DOI:** 10.3390/biom15050742

**Published:** 2025-05-21

**Authors:** Yumei Geng, Huijie Wang, Zhenzhong Bai, Rili Ge

**Affiliations:** 1 Research Center for High Altitude Medicine, Qinghai University, Xining 810001, China; yb211002010085@qhu.edu.cn (Y.G.); yb211002010090@qhu.edu.cn (H.W.); 2 Key Laboratory of High Altitude Medicine (Ministry of Education), Qinghai University, Xining 810001, China; 3 Key Laboratory of Application and Foundation for High Altitude Medicine Research in Qinghai Province (Qinghai-Utah Joint Research Key Lab for High Altitude Medicine), Qinghai University, Xining 810001, China; 4 Laboratory for High Altitude Medicine of Qinghai Province, Qinghai University, Xining 810001, China

**Keywords:** iron metabolism, HIF2α, hypoxic pulmonary hypertension, energy metabolic reprogramming, Mmitochondrial reactive oxygen species

## Abstract

Iron deficiency is highly prevalent in patients with idiopathic pulmonary hypertension; nevertheless, its role and clinical significance in hypoxic pulmonary hypertension (HPH) remain elusive. Therefore, this study aims to clarify the role and molecular mechanisms of iron in HPH. By means of a retrospective analysis of clinical data from HPH patients and examinations of HPH animal models, we discovered that both HPH patients and animal models exhibit significant iron deficiency, characterized by reduced hepatic iron storage and elevated hepcidin expression. To further explore iron’s role in HPH, we modulated iron metabolism through pharmacological and dietary interventions in chronic hypoxic animal models. The results showed that iron deficiency exacerbated chronic hypoxia-induced pulmonary hypertension and right ventricular hypertrophy, while iron supplementation alleviated these conditions. Further investigations revealed that iron regulates HIF2α expression in pulmonary arterial endothelial cells (PAECs) under chronic hypoxia. Therefore, through in vivo and in vitro experiments, we demonstrated that HIF2α inhibition attenuates chronic hypoxia-induced pulmonary hypertension and right ventricular hypertrophy. Mechanistically, chronic hypoxia-mediated iron deficiency enhances HIF2α activation, subsequently suppressing iron/sulfur cluster assembly enzyme (ISCU) expression. This leads to decreased mitochondrial complexes I and III activity, increased reactive oxygen species (ROS) production, and inhibited oxidative phosphorylation. Consequently, metabolic reprogramming in PAECs results in a proliferation/apoptosis imbalance, ultimately exacerbating hypoxia-induced pulmonary hypertension and right ventricular hypertrophy. Collectively, our findings demonstrate that iron supplementation mitigates HPH progression by modulating HIF2α-mediated metabolic reprogramming in PAECs, revealing multiple therapeutic targets for HPH.

## 1. Introduction

Pulmonary hypertension (PH) is defined as a mean pulmonary arterial pressure (mPAP) exceeding 20 mmHg at rest, measured by right heart catheterization at sea level. The elevated pulmonary artery pressure exacerbates right ventricular load, leading to right heart dysfunction and eventually right heart failure [1]. Notably, HPH is the third subgroup of PH, with a worse prognosis and higher mortality among all subgroups of PH [2]. Current therapies for PH primarily target the prostacyclin, nitric oxide (NO), and endothelin pathways. Although these pharmacological interventions provide symptomatic alleviation and confer modest survival benefits, they fail to reverse disease progression [3]. Furthermore, therapeutic drugs with the above targets are not recommended for the treatment of HPH [4]. Therefore, the identification of novel therapeutic targets for HPH has emerged as an urgent clinical imperative. The histological hallmarks of HPH are typical pulmonary vascular remodeling (PVR), manifested as increased sclerosis of proximal elastic pulmonary arterioles and muscularization of distal pulmonary arterioles [5]. Accumulating evidence demonstrates that metabolic reprogramming, characterized by enhanced aerobic glycolysis and suppressed oxidative phosphorylation, plays a pivotal role in PVR pathogenesis [6]. This tumor-like metabolic shift promotes pulmonary vascular cell proliferation and apoptosis imbalance, ultimately contributing to PH development [1,6,7,8,9,10].

Iron deficiency has been identified across various PH subtypes and is associated with adverse clinical outcomes, including elevated mPAP and reduced six-minute walk distance, both of which are responsive to iron supplementation therapy [11,12,13,14]. However, there is still a gap in the data on iron metabolism in HPH. The role of iron in HPH has been relatively little studied and is controversial [15,16,17]. Moreover, there are even fewer mechanistic studies linking iron to HPH. Iron ions are essential for synthesizing mitochondrial iron/sulfur clusters, critical cofactors of complexes I and III. These mitochondrial complexes serve as key sites for hypoxic reactive oxygen species (ROS) generation [18,19,20,21]. Therefore, we hypothesize that iron exerts indirect regulatory effects on mitochondrial ROS generation through modulation of electron transport chain components.

Hypoxia-inducible factor 2α (HIF2α), a key transcriptional regulator of chronic hypoxic adaptation, demonstrates tissue-specific expression with prominent upregulation in pulmonary endothelial cells under hypoxic conditions [22]. Beyond oxygen availability, cellular iron status represents a crucial regulator of HIF2α stability. Under conditions of hypoxia or iron depletion, diminished prolyl hydroxylase (PHD) activity prevents HIF2α ubiquitination and proteasomal degradation, facilitating its nuclear translocation and subsequent activation of downstream gene expression [23]. However, the deficiency of iron regulatory protein 2, a key protein regulating iron metabolism, can induce the activation of HIF2α [24], thereby inhibiting the biosynthesis of mitochondrial iron/sulfur clusters and the process of oxidative phosphorylation [25]. Studies show that the application of HIF2α inhibitors can not only improve the morphological characteristics of mitochondria and enhance their quality but also reshape the energy balance in cells, promoting the restoration of the energy metabolism pattern to mainly oxidative phosphorylation [26,27]. While HIF2α-mediated metabolic reprogramming has been extensively studied in oncology, its role in HPH pathogenesis remains poorly understood.

To explore the role of iron in HPH, we modulated the iron metabolism in animal models through dietary and pharmacological interventions. The results demonstrated that iron deficiency exacerbated HPH, whereas iron supplementation alleviated the progression of HPH. Both in vivo and in vitro experiments revealed that iron could regulate the expression of HIF2α in PAECs. Furthermore, we utilized small molecule inhibitors to interfere with the activity of HIF2α in vivo and in vitro and found that HIF2α inhibitors restored the PAECs’ proliferation/apoptosis homeostasis and inhibited the development of HPH by enhancing the complexes I and III expression, decreasing mitochondrial ROS generation, and remodeling the energy metabolism.

## 2. Materials and Methods

### 2.1. Patient Data

This retrospective analysis involved patients who were hospitalized in the Department of Respiratory and Critical Care Medicine of Qinghai Provincial People’s Hospital from September 2016 to September 2023. Clinical data were collected from two distinct patient cohorts: (1) chronic hypoxic lung disease patients without PH, encompassing obstructive sleep apnea-hypopnea syndrome, chronic obstructive pulmonary disease (COPD), and pulmonary interstitial fibrosis, and (2) corresponding patients with PH. Patients with PH secondary to other etiologies, including left heart disease, connective tissue disorders, and chronic thromboembolic PH, were excluded from the study. Patient enrollment was conducted through systematic screening based on the predefined inclusion/exclusion criteria, followed by comprehensive collection of clinical parameters and subsequent statistical analysis.

Serum samples were obtained from three cohorts: healthy controls without cardiopulmonary diseases, COPD patients without PH, and PH patients. HIF2α expression was quantified in all samples using a commercial ELISA kit (Cat. # CSB-E12113h, Huamei Biological Engineering Co., Ltd., Wuhan, China). 

The study protocol was approved by the Ethics Committee of Human Genetics, Ministry of Science and Technology of China (Approval No. 2023[CJ0189], date: 15 September 2023), the Institutional Review Board of Qinghai Provincial People’s Hospital (Approval No. 2023-047, date: 18 May 2023), and the Institutional Review Board of Medical College, Qinghai University (Approval No. 2022-92, date: 14 October 2022). For this retrospective analysis based on medical records, a waiver of informed consent was granted by the institutional review board. However, written informed consent was specifically obtained from all participants for blood sample collection and subsequent ELISA testing.

### 2.2. Animal Experiments

Experimental protocols and animal care in this study received approvals from the Ethics Committee of the Affiliated Hospital of Qinghai University (Approval No. P-SL-2022-058, date: 1 July 2022) and the Medical Ethics Committee of Qinghai Provincial People’s Hospital (Approval No. 2023-95, date: 20 June 2023). Male Sprague–Dawley (SD) rats (weight 200–250 g) and wild-type C57 BL/6J mice aged 8–10 weeks were purchased from Huachuang Xinnuo Pharmaceutical Science and Technology Co., Ltd. (Taizhou, China); each group was composed of 10 animals (*n* = 10).

Chronic hypoxia-induced PH model: Animals were kept in a hypobaric hypoxia compartment for 4 weeks (DYC-300, FengLei Oxygen Chamber Co., Ltd., Anshun, China). The air pressure and oxygen content align with an altitude of 5000 m above sea level. The pressure was 52.9 KPa, the O_2_ concentration was 10%, the temperature was 22.0 °C, the relative humidity was 50%, and the light and dark cycle was carried out for 12 h [28,29]. The following PH parameters were assessed after 4 weeks.

Rat Model Established by Pharmacological Intervention: Male SD rats were given deferoxamine (DFO group, 200 mg/kg; HY-B0988, MedChemExpress LLC, Monmouth Junction, NJ, USA) [30] and iron sucrose (iron sucrose group, 20 mg/kg; HY-B2068, MedChemExpress LLC, NJ, USA) intraperitoneally every other day and placed into a hypoxia or normoxia (10%) environment for 4 weeks, respectively, as previously described. Controls were given the same amount of solvent intraperitoneally.

Mouse Model Established by Dietary Intervention: Male C57 BL/6J mice were given a low iron diet (LI group, 7 mg iron/kg; M22070401, Moldiets, Biopike Technology Co., Ltd., China), a high iron diet (HI group, 2000 mg iron/kg; M22070402, Moldiets, Biopike Technology Co., Ltd., China) [15], and a standard chow (control), respectively. They were placed into a hypoxia (10%) or normoxia environment for 4 weeks, as previously described.

Mouse Model Intervened by HIF2α Inhibitor PT2385: Male C57BL/6J mice in the HCP group were kept in hypoxia (10%) for 4 weeks with daily oral gavage of PT2385 (10 mg/kg; HY-12867, MedChemExpress LLC, NJ, USA) as previously described [31]. PH parameters were assessed after 4 weeks. Mice in the NIP group were subjected to chronic hypoxic exposure (10% O_2_) for 4 weeks, followed by daily oral gavage administration of PT2385 (10 mg/kg; HY-12867, MedChemExpress LLC, NJ, USA) under normoxic conditions for an additional week. The control group was given the same amount of solvent by gavage. 

### 2.3. Transthoracic Echocardiography

Transthoracic echocardiography was utilized to evaluate right ventricular changes in animals according to previously described steps [32]. Animals were anesthetized by inhalation of isoflurane through a mask (rats: induction 3%, maintenance 2.0–2.5% in room air; mice: induction 2%, maintenance 1.0–1.5% in room air). Transthoracic echo detection was performed using the VisualSonics Vevo2100 (VisualSonics Inc., Toronto, ON, Canada) and sensor (MS-250, 13–24 MHz), and pulsed-wave Doppler ultrasound was employed to determine the pulmonary blood flow, pulmonary artery acceleration time (PAT), and pulmonary artery ejection time (PET) at the level of the aortic valve in the long-axis position. The maximal layer of the right ventricular cavity is capable of being examined in M-mode at the parasternal long-axis right ventricular outflow tract (RVOT). Right ventricular end-diastolic wall thickness (RVWT, ED) and right ventricular end-systolic wall thickness (RVWT, SD) were determined at the end-diastole and end-systole, respectively. All echocardiographic parameters were calculated using the visual Sonic Vevo770 System tool module (VisualSonics Inc., Toronto, ON, Canada), and all measurements were performed blinded by an experienced sonographer. After ultrasonography, animal models were used for hemodynamic and histological assessment.

### 2.4. Invasive Hemodynamic Measurement

Isoflurane gas anesthesia was continued after the end of ultrasound measurements in the animal model, and the right heart catheter was employed to determine the mPAP. The catheter was placed from the right exterior jugular vein, and the catheter passed via the right ventricle into the pulmonary artery. The insertion position was measured through the waveform on a bio-signal acquisition system (MP150, BIOPAC Systems, Inc., CA, USA). Following the completion of right heart catheterization, experimental animals were euthanized via cervical dislocation, with confirmation of death verified through cessation of cardiac and respiratory functions, coupled with absence of pain reflexes. The right ventricle (RV), left ventricle (LV), and septum (S) of each animal were segregated from the margin of the interventricular septum at the end of catheterization. The Fulton’s index was determined to rate the right ventricular hypertrophy based on the ratio of RV/(LV + S).

### 2.5. Animal Hematological Analysis

Blood samples were collected via cardiac puncture under anesthesia. A portion was transferred to anticoagulant tubes for complete blood count analysis. Samples were thoroughly mixed, loaded into a hematology analyzer (YAN-305A, Yuyan Scientific Instruments Co., Ltd., Shanghai, China), and analyzed using species-specific parameters. Data were exported for further processing.

### 2.6. Transmission Electron Microscope

After anesthetizing and euthanizing the rats, the heart and lungs were simultaneously removed and placed in 1 × PBS. The main pulmonary artery and its branches were carefully isolated from the rat right ventricle. Subsequently, the artery was stabilized with 3% glutaraldehyde before the post-fixation under 1% osmium tetroxide. Dehydration occurred through a graded series of acetone treatments, followed by infiltration with Epox 812 and embedding. Semithin sections underwent methylene blue staining, while Ultrathin slides were delicately sliced using a diamond knife and dual-stained with uranyl acetate and lead citrate. The slides were then meticulously observed under the JEM-1400-FLASH Transmission Electron Microscope( Japan Electron Optics Laboratory Co., Ltd., Tokyo, Japan).

### 2.7. Morphological Analysis of the Lung Vessels

The lung tissue and main pulmonary artery were fixed, embedded, and sectioned according to a generalized method, followed by hematoxylin and eosin staining (H&E), while fibrosis in the pulmonary vasculature was assessed by Masson’s trichrome staining (collagen was stained blue). The Prussian blue staining of the liver (enhanced method) was performed using a specific kit (Cat. # G1428, Solarbio Science & Technology Co., Ltd., Beijing, China). Images were acquired using a BA400 digital microscope with a digital interface (Motic China Group Co., Ltd., Xiamen, China). Finally, ImageJ software (version 1.54) was adopted to determine pulmonary artery wall thickness ratios (WT%) and pulmonary artery wall areas (WA%) as well as to assess the quantification of collagen fibers.

### 2.8. Immunohistochemical Assay

Immunohistochemical experiments were performed using paraffin-embedded sections, deparaffinized with xylene and diluted with ethanol. Next, 0.01 M sodium citrate buffer solution (pH 6.0) was heated in a water bath to 95 °C. The tissue sections were then incubated in this buffer at 95 °C for 20 min. After the buffer cooled down naturally, the sections were retrieved, thus completing the antigen-repair process. After blocking the pulmonary vascular sections with an immunostaining blocking solution at room temperature for 1 h, tissue sections were sequentially incubated with primary antibodies: rabbit anti-Hepcidin monoclonal antibody (1:500; Cat. # ET1704-22; Hua’an Biotechnology Co., Ltd., Hangzhou, China) and rabbit anti-HIF-2α polyclonal antibody (1:100; Cat. # PA1-16510; Thermo Fisher Scientific, Waltham, MA, USA) overnight at 4 °C, followed by horseradish peroxidase (HRP)-conjugated secondary antibodies (1:100; Cat. # GB2003; Servicebio Technology Co., Ltd., Wuhan, China) for 60 min at room temperature. Then, DAB was used to develop the color, hematoxylin was used to re-stain the nuclei, and neutral gum was used to seal the film. A BA400 digital microscope (Motic China Group Ltd., Xiamen, China) was used for observation, with ImageJ software utilized to quantify the HIF2α expression.

### 2.9. Immunofluorescence Assay

For the immunofluorescence assay in animal models, pulmonary vascular sections underwent dewaxing and dehydration, followed by the antigenic repair procedure. The citrate buffer (pH 6.0) was heated to 95 °C in a water bath. The tissue sections were then incubated in this buffer at 95 °C for 20 min. After natural cooling of the buffer, the sections were removed. Following incubation with the immunostaining blocking solution at room temperature for 1 hour, HIF2α (1:100, Cat. # PA1-16510, Invitrogen Corporation, CA, USA), smooth muscle actin (SMA, 1:100, Cat. # A2547, Sigma-Aldrich Co. LLC., MO, USA), and glucose transporter 1 (GLUT1, 1:100, Cat. # ab115730, Abcam public limited Company, Cambridge, UK) antibodies were added and samples incubated overnight at 4 °C. Following three routine washes with 1× PBS, the slides were incubated with a fluorescent secondary antibody at room temperature for 1 h. The nuclei were re-stained with DAPI, and the image was examined through a confocal laser microscope (FV1000, Olympus Corporation, Tokyo, Japan). For the immunofluorescence assay in the cellular model, the treated PAECs were fixed, permeabilized, and blocked following standard procedures. They were then incubated with the primary antibody Caspase 3 (1:1000, Cat. # 19677-1-AP, Proteintech Biotechnology Co., Ltd., Wuhan, China) at 4 °C overnight. Subsequently, they were incubated with Alexa Fluor™ 488 (1:1000; Cat. # ab150077, Abcam public limited Company, Cambridge, UK) and DAPI in sequence. For the detection of mitochondrial membrane potential (MMP) and apoptosis, mitochondrial superoxide (MitoSOX) fluorescence assays were performed according to the kit instructions (Cat. # C1071M, Beyotime Biotechnology Co., Ltd., Haimen, China; Cat. # S0061M, Beyotime). The images were examined through a fluorescence microscope (THUNDER imager, Leica Microsystems GmbH, Wetzlar, Germany), and finally, the immunofluorescence intensity of protein signals was analyzed semi-quantitatively by ImageJ software.

### 2.10. Quantitative Real-Time Polymerase Chain Reaction (qPCR) Analysis

Total RNA was extracted from the liver tissue samples using an RNA extraction reagent (Cat. # G3013; Servicebio, China). After tissue homogenization, the mixture was centrifuged, and the supernatant was processed with a chloroform substitute followed by isopropanol precipitation. The RNA pellet was washed with 75% ethanol, air-dried, and dissolved in an appropriate volume of RNase-free water. RNA concentration and purity were determined using a spectrophotometer. Subsequently, reverse transcription was carried out with a reverse transcription kit (Cat. # G3337; Servicebio, China) in a reaction mixture containing total RNA, gDNA remover, and a 5× reverse transcription supermix. The reverse-transcribed cDNA was then used as a template for quantitative PCR. The qPCR reaction system included 2× Universal Blue SYBR Green qPCR Master Mix (Cat. # G3326; Servicebio, China), gene-specific primers, cDNA, and nuclease-free water. The reaction was performed in triplicate in a 0.1 ml PCR reaction plate, which was sealed with a PCR sealing film and centrifuged. PCR amplification was conducted on a fluorescence-quantitative PCR instrument with pre-denaturation at 95 °C for 30 s, 40 cycles of denaturation at 95 °C for 15 s, and annealing/extension at 60 °C for 30 s, followed by melt/curve analysis from 65 °C to 95 °C with fluorescence signal collection at 0.5 °C intervals (CFX Series Real-Time PCR Detection Systems, Bio-Rad Laboratories, Inc., CA, USA). Finally, relative gene expression was calculated using the ΔΔCT method. The gene-specific primer sets were listed as follows: Hepcidin, 5’-GACAGAAGGCAAGATGGCACTA-3’; 5’-GTCTCGCTTCCTTCGCTTCA-3’; GAPDH, 5’-CTGGAGAAACCTGCCAAGTATG-3’; 5’-GGTGGAAGAATGGGAGTTGCT-3’.

### 2.11. Western Blot Analysis

Proteins were extracted from lung tissues through RIPA lysis buffers and quantified using a BCA kit (Cat. # A55864, Thermo Fisher Scientific, MA, USA). First, 20 µg protein samples of each group were run on SDS-PAGE (6%-10%) and subsequently transferred to a polyvinylidene fluoride membrane (PVDF, 0.22 μm, Merck Millipore GmbH, Darmstadt, Germany). After protein transfer, the PVDF membranes were blocked with 5% skimmed milk for 1 h at room temperature, followed by overnight incubation at 4 °C with a suitable primary antibody (total oxidative phosphorylation Rodent WB antibodies, 1:1000, Abcam, Cat. # ab110413, USA; HIF2α, 1:1000, Cat. # A7553, Abclonal; GLUT1, 1:1000,Cat. # HY-P80494, MCE; lactate dehydrogenase [LDH], 1:1000, Cat. # HY-P80494, Proteintech; iron/sulfur cluster assembly enzyme [ISCU], 1:500, Cat. # 14812-1-AP, Proteintech; β-actin, 1:5000, Cat. # HY-P80494, Proteintech; Glyceraldehyde-3-Phosphate Dehydrogenase [GAPDH], 1:5000, Cat. # 10494-1-AP, Proteintech). After incubation with secondary antibodies for 1 h at room temperature, chemiluminescence signals were detected using an ECL kit (Cat. # BMU102, Abbkine Scientific Co., Ltd., Wuhan, China). Finally, ImageJ software was employed to analyze the intensity of the bands.

### 2.12. Non-Targeted Metabolomics Analysis Based on Liquid Chromatography-Mass Spectrometry (LC-MS)

Analyzing metabolites in mouse lung tissue samples involved several steps to ensure accurate and comprehensive results. Initially, the tissue samples were weighed before being lyophilized and ground in a single tube with a tungsten bead using a grinding mill (65 Hz, 1 min). Metabolites were obtained by extraction with a mixture of acetonitrile, methanol, and water, followed by ultrasonic treatment for 1 hour and centrifugation (4 °C, 14,000× *g*, 20 min). The resulting supernatants were evaporated to dryness under vacuum. Metabolomic profiling was performed with a UPLC-ESI-Q-Orbitrap-MS system, consisting of a Shimadzu Nexera X2 LC-30AD UHPLC (Shimadzu Corporation, Kyoto, Japan) and a Thermo Scientific Q-Exactive Plus (Thermo Fisher Scientific Inc., MA, USA). Liquid chromatography segregation was implemented through an ACQUITY UPLC^®^ HSS T3 column with a specific gradient of mobile phases A and B. The ESI source conditions were optimized for positive and negative ion mode MS data collection. The instrument was used for scanning a range of *m*/*z* values for full MS, with resolutions of 70,000 and 17,500 at different *m*/*z* values for MS and MS/MS scans, respectively. The raw MS was shown through the MS-DIAL software (version 5.0) for peak alignment, retention time rectification, and peak region extraction. Metabolites were identified based on precise mass and MS/MS information by comparing them with various public databases. Only metabolites with significant quantitative values were retained for analysis. Overall, this rigorous analytical approach allowed for the comprehensive profiling and identification of metabolites in mouse lung tissue samples. The use of advanced equipment and data processing methods ensured the accuracy and reliability of the results obtained in this study.

Utilizing R (v 4.0.3) and its associated packages, comprehensive multivariate analysis and modeling were conducted. Prior to modeling, the data underwent mean-centring with Pareto scaling. Three key modeling techniques were employed: principal component analysis (PCA), orthogonal partial least-squares discriminant analysis (OPLS-DA), and partial least-squares discriminant analysis (PLS-DA). The risk of overfitting was rigorously assessed using permutation tests for all evaluated models. The descriptive models were measured by cumulative R2X and R2Y values, with perfect scores of 1 indicating a complete explanation of variance. Prediction performance, on the other hand, was evaluated by cumulative Q2 values (perfect model: Q2 (cum) = 1) and permutation tests (*n* = 200). In permuted models, the values of R2 and Q2 at the Y-axis intercept were required to be below those of non-permuted models, ensuring predictive validity (Supplemental File: Table S1, Figure S1A,B). OPLS-DA specifically enabled the recognition of discriminatory metabolites through the variable significance on the projection (VIP) score. This mark quantifies each variable’s contribution to distinguishing between sample classes. VIP values are calculated as a weighted sum of squares of PLS weights. Values above 1.5 were typically considered remarkable, denoting a robust discriminatory ability. Consequently, these scores serve as a critical criterion for biomarker selection.

Discriminating metabolites were recognized through a significant VIP threshold from the OPLS-DA model and a two-tailed Student’s *t*-test on standardized initial data. ANOVA was conducted for multiple groups, and metabolites showing VIP values exceeding 1.0 and *p*-values below 0.05 were deemed statistical significance. Fold change was calculated by taking the logarithm of the average mass spectrometric response ratio between two arbitrary classes. Furthermore, the identified differential metabolites were clustered using R packages for further analysis.

The study analyzed differential metabolite data to identify disrupted biologic pathways with the KEGG database (http://www.kegg.jp, accessed on 23 September 2023). KEGG enrichment analyses, including Fisher’s exact test and FDR correction, were conducted to determine statistically significant changes. Enriched KEGG pathways were discovered to be significant at a level of *p* < 0.05. This approach helped to pinpoint pathways affected by perturbations in biological processes.

### 2.13. Targeted Metabolomics Analysis

Mouse lung tissue was weighed prior to the extraction of metabolites. These tissues were then dried and ground within a 2 mL Eppendorf tube using a grinding mill (1 min, 65 Hz). Metabolites were obtained through a 1 mL mixture of acetonitrile, methanol, and water (2:2:1) that had been pre-cooled and subsequently subjected to 1 hour of ultrasonic treatment in ice baths. After this, the mixture was cooled for another hour at −20 °C and then centrifuged (4 °C, 14,000× *g*, 20 min). Accordingly, the supernatants were collected, evaporated to dryness under vacuum, and dissolved in 50% acetonitrile. To guarantee the data quality obtained from metabolic profiling, quality control (QC) specimens were prepared by combining aliquots from all the specimens to make one representative sample pool. The QC specimens underwent identical analysis as the experimental specimens for data normalization. After preparation, the dried extracts were filtered through a 0.22 µm cellulose acetate filter, transferred to 2 mL HPLC vials, and stored at −80 °C until further analysis. This meticulous process was critical in ensuring accurate and reliable results in metabolic profiling studies.

The LC/MS analysis utilized a Shimadzu Nexera X2 LC-30AD system with an ACQUITY UPLC BEH Amide column as well as a triple quadrupole mass spectrometer (QTRAP 5500, AB SCIEX LLC, MA, USA). Metabolites were identified using electrospray ionization in both negative and positive modes. Samples of 2 μL were injected into the LC autosampler. The column at 45 °C had a flow rate of 300 μL/min. One gradient elution method was employed, consisting of 20 mM ammonium acetate and 5% acetonitrile with a pH of 9.45 (solvent A) and 100% acetonitrile (solvent B). The gradient started at 5% solvent A for 1 min, increased to 45% solvent A over 12 min, then to 60% solvent A over 1 min, with a 2-min hold followed by returning to the initial conditions within 0.1 min, followed by a 3-min re-equilibration. QC specimens were interspersed every six to eight specimens in the analysis process.

The mass spectrometer was operated under specific conditions. A dwell time of 200 ms was used for optimal performance. A metabolite MRM library was developed by analyzing each standard metabolite at 50 mg/mL concentration using LC-MS/MS to determine the best MRM transition parameters. The retention times for 40 energy-related metabolites were established by measuring their individual MRM transitions (Q1/Q3). Supplemental File (Table S2 and Figure S1C) depict the precursor/product ion pairs for these targeted energy metabolites. A reference standard containing the 40 metabolites was then prepared in a serial dilution for analysis using LC-MS.

MultiQuant software (version 3.1) was utilized to process the raw MRM data to conduct peak discoveries, alignment, extraction, and filtration. Through a significant threshold of fold change (FC) and two-tailed Student’s *t*-test (*p*-value) on the standardized raw data, discriminating metabolites were identified. A one-way analysis of variance (ANOVA) was performed, with the intention of determining the *p*-value for the analysis on multiple groups. Metabolites showing an FC over 1.0 and a *p*-value below 0.05 were deemed statistically significant. The FC was determined as the logarithm of the mean mass response (area) ratio between two arbitrary classes. Subsequently, cluster analyses were implemented through the R package based on the identified differential metabolites. Various models, including principal component analysis (PCA), orthogonal partial least squares-discriminant analysis (PLS-DA), and partial least squares-discriminant analysis (OPLS-DA), were built in accordance with the non-targeted metabolomics approach. Detailed parameters can be discovered in Supplemental File: Table S3 and Figure S1D,E.

### 2.14. Measurement of ATP, Lactic Acid, and NO Content

ATP and lactate levels in serum and lung tissue were assessed by an ATP Assay Kit (Cat. # G4309-48T, Servicebio, Wuhan, China) and a Lactate Assay Kit (Cat. # A019-2-1, Jiancheng Bioengineering Institute, Nanjing, China) following the producer’s guidelines. The NO content in animal serum and cell culture medium was measured using specific kits (Cat. # A012-1-2, Jiancheng Bioengineering Institute, Nanjing, China) according to the instructions. 

### 2.15. Quantification of Iron Metabolism Parameters

Serum and tissue iron, as well as ferritin, were measured according to the instructions of the reagent kit. The kit for iron measurement was from Nanjing Jiancheng Bioengineering Institute, China (Cat. # A039-1-1; A039-2-1), and the kit for ferritin measurement was from Elabscience Biotechnology Co., Ltd. (Wuhan, China) (Cat. # E-EL-R3018).

### 2.16. Cell Culture

Rat pulmonary artery endothelial cells (RPAECs, CD31 fluorescence identification is reported in Supplementary Figure S1F) and human pulmonary artery endothelial cells (HPAECs, the short tandem repeat (STR) assay report is shown in Supplementary Figure S1G,H) were supplied by Wuhan Procell Life Technology Co., Ltd., (Wuhan, China) and Bena Biological Technology Co., Ltd. (Xinyang, China)., respectively, and cultured with endothelial cell medium (ECM, ScienCell Research Laboratories, CA, USA) containing vascular endothelial growth factor at 37 °C with 5% CO_2_. The cells at passages 3–5 were used in this study. PAECs cultured under hypoxia were treated with 1% O_2_ concentration for 24–48 h [33,34].

### 2.17. Cellular Pharmacological Interventions

RPAECs were treated with DFO (100 µmol/L, Cat. # HY-B0988, MedChemExpress LLC, NJ, USA), iron sucrose (50 µmol/L, Cat. # HY-B2068, MCE), and PT2385 (20 µmol/L, Cat. # HY-12867, MCE) as previously described [35,36].

HPAECs were treated with PT2385 (20 µmol/L, Cat. # HY-12867, MCE) and Mitoquinone mesylate (MitoQ, 1 µmol/L, Cat. # HY-100116A, MedChemExpress LLC, NJ, USA) for 24–72 h (Figure S2A) [37].

### 2.18. Cell Proliferation and Migration Experiment

A CCK-8 kit (Cat. # E-CK-A362, Elabscience) was employed to investigate cell proliferation, as per the producer’s guidelines. A microplate reader (BioTek Instruments, Inc., Vermont, USA) was employed to measure the absorbance at 450 nm. Detection of cell proliferation was performed in light of the instructions of the EDU Cell Proliferation Kit (Cat. # C0071L, Beyotime), and image observation was performed using a fluorescence microscope (ECHO/RVL-100). Then, ImageJ software was employed to analyze the data. For transwell experiments, after starvation pretreatment, PAECs were inoculated in the upper compartment of 24-well transwell plates, with ECM containing 10% fetal bovine serum inoculated in the lower compartment. PAECs were continuously migrated for 24 h under normoxia or hypoxia, respectively, immobilized in 4% paraformaldehyde, and subsequently stained with 1% crystal violet (30 min at room temperature). A Zeiss Axio Vert A1 microscope (Carl Zeiss AG, Oberkochen, Germany) was used to record cell images in four random fields of view, and finally, the cells were counted by ImageJ software. For wound healing experiments, PAECs were first cultured until they reached confluence and then scratched with a 200 μL pipette tip. Afterward, the cells were incubated in the serum-free medium in a normoxic or hypoxic environment for another 72 h. A Zeiss Axio Vert microscope was used to take photographs at 0, 24, 48, and 72 h, and ImageJ software was used to assess the area of the wound and to calculate the percentage of wound healing.

### 2.19. Seahorse Experiment

Seahorse XFe 96 (Agilent Technologies, Inc., CA, USA) was utilized to detect the extracellular acidification rate (ECAR) and mitochondrial oxygen consumption rate (OCR) of PAECs. Sensor cartridges were hydrated by adding 200 μL calibration solution per well in utility plates 24 h prior to experiments and incubated overnight at 37 °C under CO_2_-free conditions. PAECs (1 × 10⁴ cells/well) were seeded in 96-well Seahorse XF plates and treated with drugs under specified oxygen concentrations for 24 h. Culture medium was replaced with Seahorse XF assay medium (5 mM glucose, 2 mM pyruvate), followed by a 40-min pre-incubation at 37 °C (CO_2_-free) for pH/temperature equilibration. The following compounds were loaded into cartridge ports: oligomycin (2 μM, Port A), FCCP (0.5 μM, Port B), rotenone/antimycin A (0.5 μM, Port C). Hydrated cartridges and cell plates were loaded into the analyzer for automated calibration. OCR/ECAR measurements were performed per manufacturer’s protocols, with cycle parameters (interval timing, mixing/measurement duration) defined in Seahorse XF software (version 2.6.1.53). Data were exported for statistical analysis.

### 2.20. Measurement of Mitochondrial Complex I and III Activities 

Cells were seeded uniformly in T25 flasks. Following complete adherence, pharmacological interventions under defined oxygen tensions were applied for 24 h prior to cell collection. Mitochondrial complex I/III activities were subsequently quantified per manufacturer’s protocols (Cat. # E-BC-K834-M and E-BC-K836-M; Wuhan Elabscience Biotechnology Co., Ltd.).

### 2.21. Statistical Analysis

The whole data were processed by SPSS 28.0 and GraphPad 10.0 software. The data with normal distribution were denoted as the mean ± standard deviation, with the skewed distribution shown as the median (the first quartile and the third quartile). The two groups were compared through an independent-sample *t*-test for normal distribution data and a non-parametric Mann–Whitney test for skewed distribution data. One-way ANOVA and LSD post hoc tests were adopted to contrast multiple groups, with Spearman correlation utilized to explore the relationship between disparate variables. A *p*-value of less than 0.05 was considered statistically significant.

## 3. Results

### 3.1. Iron Deficiency Is Present in Patients with PH Due to Chronic Hypoxic Lung Disease

In total, 169 patients were retrospectively analyzed, of whom 104 patients had chronic hypoxic lung disease combined with HPH, and 65 patients had chronic hypoxic lung disease without HPH. The detailed clinical data are shown in Supplemental File: Table S4. With regard to patients suffering from chronic hypoxic lung disease with HPH, serum iron and transferrin saturation (TSAT) were lower than those suffering from chronic hypoxic lung disease without HPH (Figure S1J). Two models were chosen to define iron deficiency better. One was based on the currently accepted definition of iron deficiency by researchers, i.e., serum iron < 10 μmol/L accompanied by TSAT < 20% in males and <15% in females [38]. The iron deficiency rate was found to be 37% among patients suffering from chronic hypoxic lung disease with HPH, which was remarkably higher than that in patients with chronic hypoxic lung disease without HPH (Figure S1I). The other model was based on recent studies that defined TSAT < 21% as iron deficiency [11]. The results suggested that the iron deficiency rate was 56% among patients suffering from chronic hypoxic lung disease with HPH and 28% in patients suffering from chronic hypoxic lung disease without HPH (Figure S1I). Regardless of the defining model of iron deficiency, patients in the iron deficiency group had higher pulmonary arterial systolic pressure (PASP) than patients with iron sufficiency(Figure S1K). 

### 3.2. Iron Metabolism Disorders Under Chronic Hypoxia: Iron Chelator Aggravates While Iron Sucrose Alleviates Hypoxia-Induced PH in Rats

**Figure 1:**
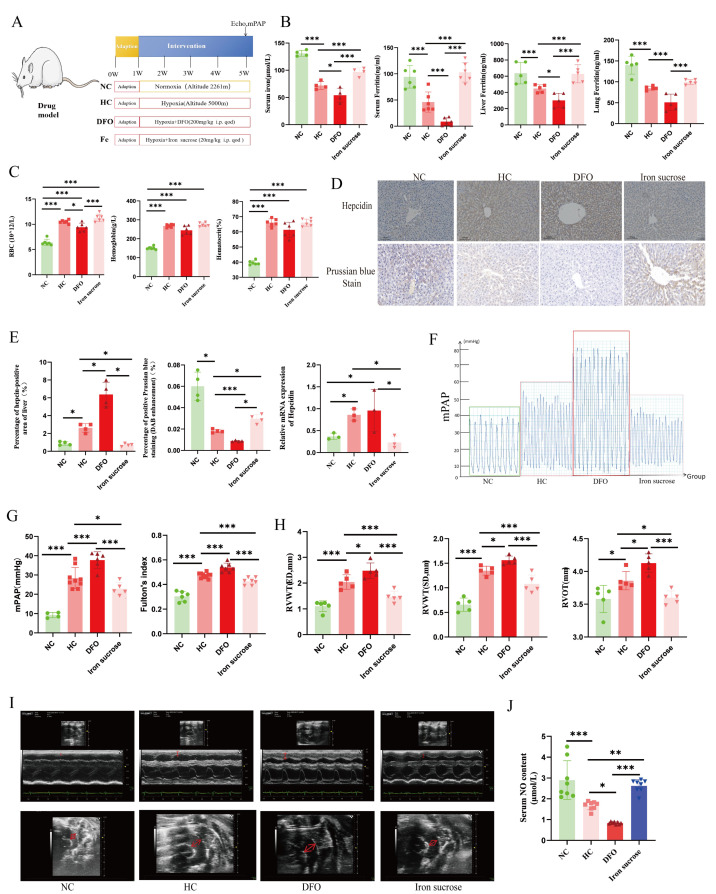
Iron chelator aggravates hypoxia-induced PH, and iron supplementation alleviates hypoxia-induced PH. (**A**) Graphical summary of animal modeling. (**B**) Assessment of iron metabolism parameters in animal models. (**C**) Analysis of erythroid parameters in rat models with iron intervention under chronic hypoxia. (**D**) Images of the Hepcidin immunohistochemistry (scale bar, 100 μm, magnification, ×100) and Prussian blue enhancement staining of the liver (scale bar, 20 μm, magnification, ×400). (**E**) The quantification results of the Hepcidin immunohistochemistry, Prussian blue enhancement staining, and mRNA expression of the liver. (**F**) Images of the mPAP waves. (**G**) Summary data of mPAP and Fulton’s index. (**H**,**I**) Echocardiographic quantification and images of RVWT, and RVOT (The red arrows in the upper panel of (**I**) indicate RVWT; those in the lower panel denote RVOT width). (**J**) Assessment of NO content in rat serum. NC: Normoxia control group; HC: Hypoxia control group; DFO: Hypoxia combined with deferoxamine group; Iron sucrose: Hypoxia combined with iron sucrose group; mPAP: Mean pulmonary artery pressure; RVWT, SD: Right ventricular end-systolic wall thickness; RVWT, ED: Right ventricular end-diastolic wall thickness; RVOT: Right ventricular outflow tract. In the statistical graph, each point represents the number of experimental animals. The data of the animal model are presented as mean ± SD; * *p* < 0.05; ** *p* < 0.01; *** *p* < 0.0001.

To investigate the function of iron in HPH, we exposed a rat model to a chronic hypoxia environment for 4 weeks to create a model of HPH and administered drugs to modulate iron metabolism in the model (Figure 1A). The results showed that chronic hypoxia resulted in a decrease in serum iron and ferritin concentrations of animal models, accompanied by a decrease in hepatic tissue ferritin and a decrease in hepatic tissue iron content (Figure 1B,D,E). Furthermore, the mRNA and protein expression of Hepcidin, an essential factor regulating iron metabolism, was elevated in the liver under hypoxia (Figure 1D,E). Chronic hypoxia elevated red blood cell (RBC) count, hemoglobin, and hematocrit. Iron chelators attenuated hypoxia-induced erythroid hyperplasia, while iron sucrose exhibited no modulatory effect on hypoxic erythropoiesis (Figure 1C). Interestingly, iron deficiency exacerbated hypoxia-induced elevation of mPAP and Fulton’s index (Figure 1F,G) and worsened the indices of right ventricular structure and function, such as RVWT, PAT/PET, and RVOT (Figure 1H,I), which could be ameliorated by iron supplementation. To further assess the effect of iron on endothelial cell function, we measured serum NO levels. The results revealed that iron sucrose was effective in reversing the reduction in NO due to chronic hypoxia (Figure 1J). 

**Figure 2:**
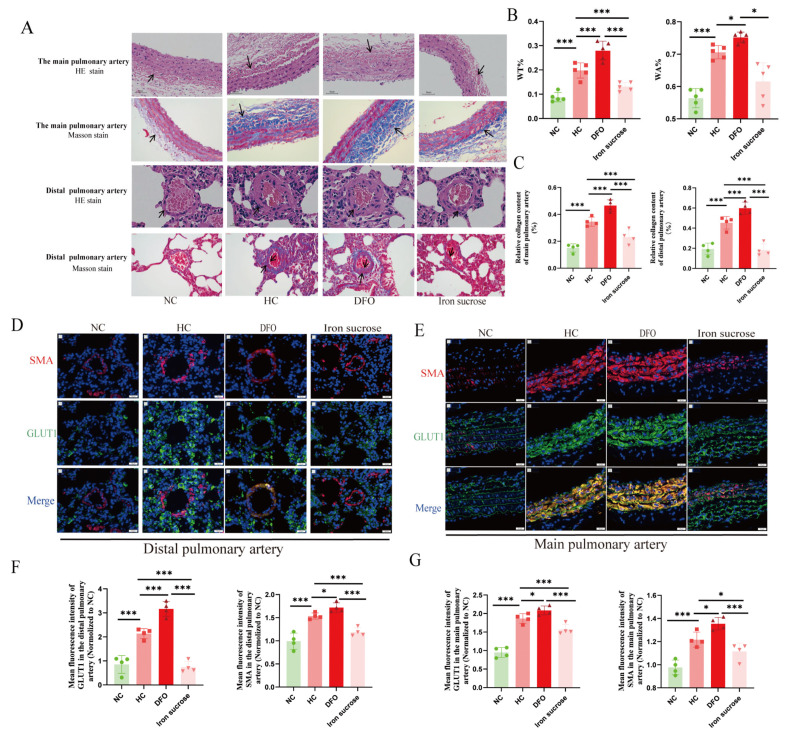
Iron chelator aggravates hypoxia-induced PVR, and iron supplementation alleviates hypoxia-induced PVR. (**A**–**C**) The representative images and summary data of HE (The main pulmonary artery, scale bar, 50 μm, magnification, ×200; Distal pulmonary artery, scale bar, 20 μm, magnification, ×400) and Masson staining (The main pulmonary artery, scale bar, 50 μm, magnification, ×200; Distal pulmonary artery, scale bar, 20 μm, magnification, ×400) in rats’ lung sections, in HE-stained main pulmonary arteries, arrows indicate outer collagen fibers (stained blue in Masson staining); in distal pulmonary arteries, HE arrows denote vascular wall thickness, with fibrotic walls stained blue in Masson staining. (**D**–**G**) The representative images (scale bar, 20 μm, magnification, ×400) and summary data of immunofluorescence in rats’ pulmonary vessels. NC: Normoxia control group; HC: Hypoxia control group; DFO: Hypoxia combined with deferoxamine group; Iron sucrose: Hypoxia combined with iron sucrose group; WT%: Pulmonary artery wall thickness ratio; WA%: Pulmonary artery wall areas ratio; SMA: α-smooth muscle actin; GLUT1: Glucose transporter 1. In the statistical graph, each point represents the number of experimental animals. The data of the animal model are presented as mean ± SD; * *p* < 0.05; *** *p* < 0.0001.

PVR is a crucial pathogenesis of HPH, so we explored the role of iron in PVR. HE staining of the pulmonary vasculature in drug-intervened rats suggests hypoxia-induced vascular occlusion and muscularization of the distal pulmonary artery (Figure 2A,B). In addition, Masson staining showed an increase in collagen fibers in the external layer of the pulmonary aorta and in the internal and external layers of the distal pulmonary arteries (Figure 2A,C). GLUT1 facilitates cellular glucose uptake, while SMA marks pulmonary vascular muscularization. Immunofluorescence analysis revealed hypoxia-induced upregulation of GLUT1 and SMA in pulmonary arteries and distal arterioles, which were ameliorated by iron supplementation (Figure 2D–G).

### 3.3. Iron-Deficient Diet Exacerbates PH, and Iron-Enriched Diet Attenuates PH Under Chronic Hypoxia in a Mouse Model

**Figure 3:**
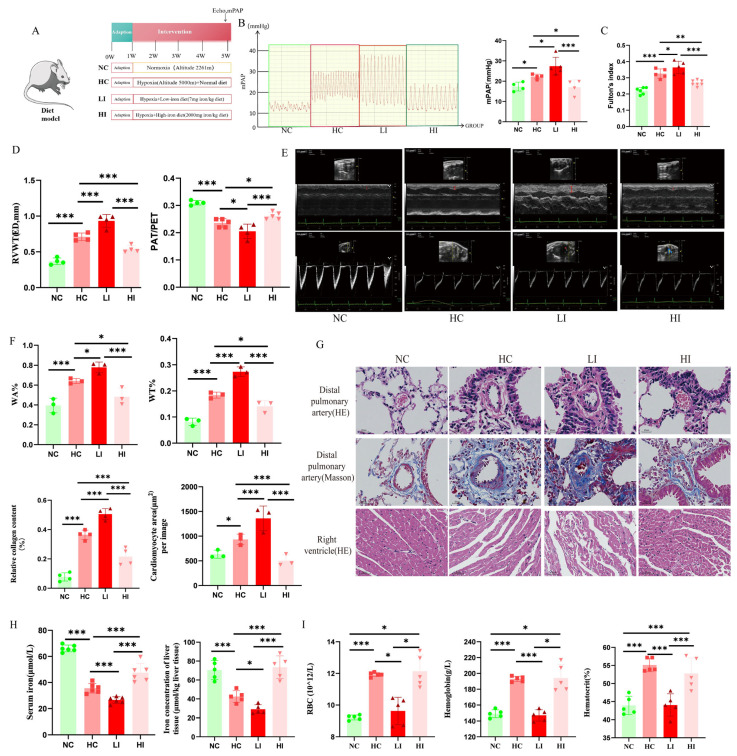
Iron-deficient diet aggravates PH, and iron-enriched diet attenuates PH under chronic hypoxia in a mouse model. (**A**) Graphical summary of animal modeling. (**B**) Images and summary data of the mPAP waves. (**C**) Summary data of the Fulton’s index. (**D**,**E**) Summary data and echocardiographic images of RVWT(ED) and PAT/PET (The red arrows in the upper panel of (**E**) indicate RVWT). (**F**,**G**) The summary data and representative images of HE and Masson staining in the mouse lung and right ventricular sections (For HE and Masson staining of distal pulmonary arteries, scale bar, 20 μm, magnification, ×400; for myocardial HE staining, scale bar, 50 μm, magnification, ×200). (**H**) Assessment of iron metabolism parameters in animal models. (**I**) Assessment of erythroid parameters in animal models. NC: Normoxia control group; HC: Hypoxia control group; LI: Hypoxia combined with iron-deficient diet group; HI: Hypoxia combined with iron-enriched diet group; mPAP: Mean pulmonary artery pressure; RVWT, ED: Right ventricular end-diastolic wall thickness; PAT/PET: Pulmonary artery acceleration time/pulmonary artery ejection time; WT%: Pulmonary artery wall thickness ratio. In the statistical graph, each point represents the number of experimental animals. The data of the animal model are presented as mean ± SD; * *p* < 0.05; ** *p* < 0.01; *** *p* < 0.0001.

To further validate the role of iron in HPH, we utilized dietary intervention for iron metabolism in a mouse model (Figure 3A). The results showed that a long-term iron-deficiency diet aggravated chronic hypoxia-induced iron deficiency and PH, and an iron-enriched diet attenuated chronic hypoxia-induced iron deficiency and PH in mice (Figure 3B,H). Echocardiography and Fulton’s index data showed that a long-term iron deficiency diet aggravated chronic hypoxia-induced right ventricular hypertrophy, and an iron-enriched diet attenuated chronic hypoxia-induced right ventricular hypertrophy (Figure 3C–E). HE staining of the pulmonary vasculature suggested hypoxia-induced vascular occlusion and muscularization of the distal pulmonary artery (Figure 3F,G). The morphological assessment of the myocardium also showed that chronic hypoxia disturbed the arrangement of cardiomyocytes, thickened myocardial fibers, enlarged the nuclei of some cardiomyocytes, and increased interstitial myocardium. Masson staining also showed that chronic hypoxia induced an increase in collagen fibers in the internal and external layers of the distal pulmonary arteries (Figure 3F,G), which could be improved by an iron-enriched diet. Furthermore, under chronic hypoxic conditions, a long-term iron-deficient diet suppresses erythroid production (including RBC, hemoglobin, and hematocrit), whereas an iron-sufficient diet exhibits no significant effect on hypoxia-induced erythropoiesis (Figure 3I). 

### 3.4. Iron Status Modulates HIF2α Activity In Vivo and In Vitro Under Chronic Hypoxia

**Figure 4:**
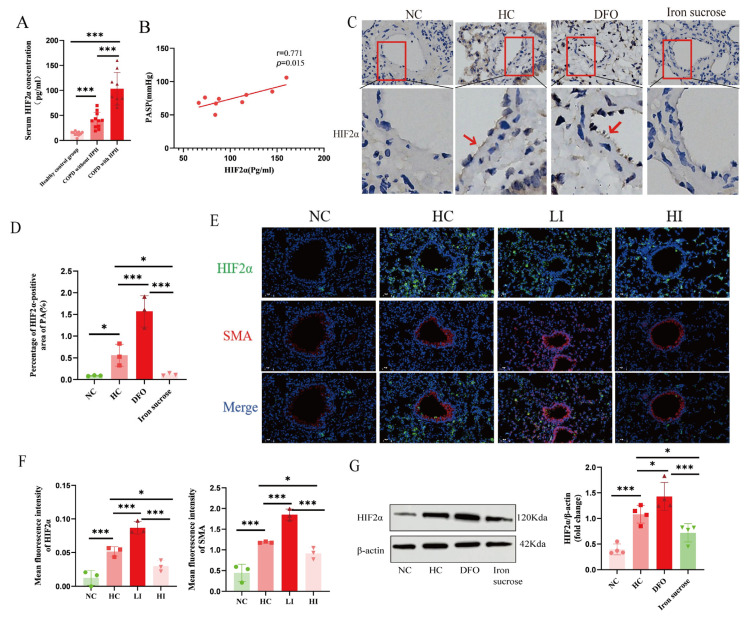
Iron status modulates HIF2α activity in vivo. (**A**) Serum HIF2α expression in patients with or without HPH. (**B**) The correlation analysis of serum HIF2α and PASP. (**C**,**D**) Immunohistochemistry quantification and representative images of rats’ pulmonary vessels, red arrows in (**C**) indicate HIF2α-positive staining (scale bar, 20 μm, magnification, ×400). (**E**,**F**) Immunofluorescence quantification and representative images (scale bar, 20 μm, magnification, ×400) of rats’ pulmonary vessels. (**G**) Western blot assessment of HIF2α protein expression in rats’ lung tissues. NC: Normoxia control group; HC: Hypoxia control group; LI: Hypoxia combined with iron-deficient diet group; HI: Hypoxia combined with iron-enriched diet group; DFO: Hypoxia combined with deferoxamine group; Iron sucrose: Hypoxia combined with iron sucrose group; SMA: α-smooth muscle actin; The original images of Western Blot can be found in the Supplementary Files. In the statistical graph, each point represents the number of patients or experimental animals. The data are presented as mean ± SD; * *p* < 0.05; *** *p* < 0.0001.

It is well known that iron, as an essential cofactor for PHD, is involved in the regulation of HIFs’ activity. Therefore, we detected HIFs expression by Western blot. We did not find HIF1α bands that appeared at the corresponding locations, and referring to previous articles that reported that HIF2α under chronic hypoxia is the predominant protein that mediates chronic hypoxia adaptation, we examined HIF2α again. Intriguingly, we detected a significant increase in HIF2α expression under chronic hypoxia (Figure 4G). Furthermore, we detected HIF2α expression in the serum of patients enrolled in the study with HPH and found that serum HIF2α in patients with COPD and HPH patients was higher than that in patients with COPD but without HPH and in the healthy control group (Figure 4A), and serum HIF2α expression was highly positively correlated with PASP (Figure 4B). Meanwhile, to further clarify whether iron affects the expression of HIF2α, immunohistochemistry and immunofluorescence were performed in animal models (Figure 4C–F), and we found that iron supplementation could inhibit the hypoxia-induced increase in HIF2α expression of the pulmonary vasculature. 

**Figure 5:**
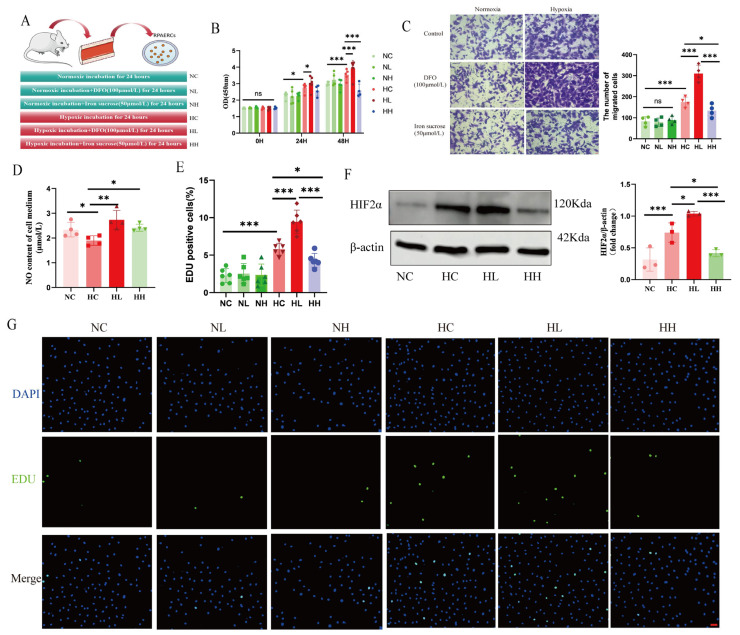
Iron status modulates HIF2α activity in vitro. (**A**) Graphical representation of detailed experimental methods for iron intervention in RPAECs. (**B**) The summary data of CCK8 assay. (**C**) The representative images and summary data of the Transwell assay, scale bars = 50 μm. (**D**) The summary data of NO content in RPAECs. (**E**,**G**) The summary data and images of EDU assay, scale bars = 50 μm. (**F**) Western blot assessment of HIF2α protein expression in RPAECs. NC: Normoxia control group; NL: Normoxia combined with deferoxamine group; NH: Normoxia combined with iron sucrose group; HC: Hypoxia control group; HL: Hypoxia combined with deferoxamine group; HH: Hypoxia combined with iron sucrose group; NO: Nitric oxide. The original images of Western Blot can be found in the Supplementary Files. In the statistical graph, each point represents the number of experimental repetitions. The data are presented as mean ± SD; ns: No statistical significance; * *p* < 0.05; ** *p* < 0.01; *** *p* < 0.0001.

To further validate the regulatory effect of iron status on HIF2α activity, we performed relevant in vitro experiments on RPAECs (Figure 5A). The data in vitro showed that RPAECs developed hyperproliferative and migratory phenotypes under chronic hypoxia (Figure 5B,C,E,G), with a decrease in NO production (Figure 5D). Treatment with different iron intervention to RPAECs under normoxia had no effect on the phenotype of RPAECs. However, under hypoxia, iron sucrose ameliorated the phenotypes of RPAEC hyperproliferation and migration (Figure 5B,C,E,G). Western blot experiments on RPAECs also showed that iron status modulated HIF2α activity in vitro (Figure 5F). On the whole, the findings demonstrate that iron supplementation modulates the HIF2α activity of RPAECs to improve hypoxia-induced endothelial dysfunction and ameliorates hypoxia-induced PH. 

### 3.5. HIF2α Inhibition Remodels Endothelial Cell Phenotype Under Hypoxia and Attenuates Chronic Hypoxia-Induced PH

**Figure 6:**
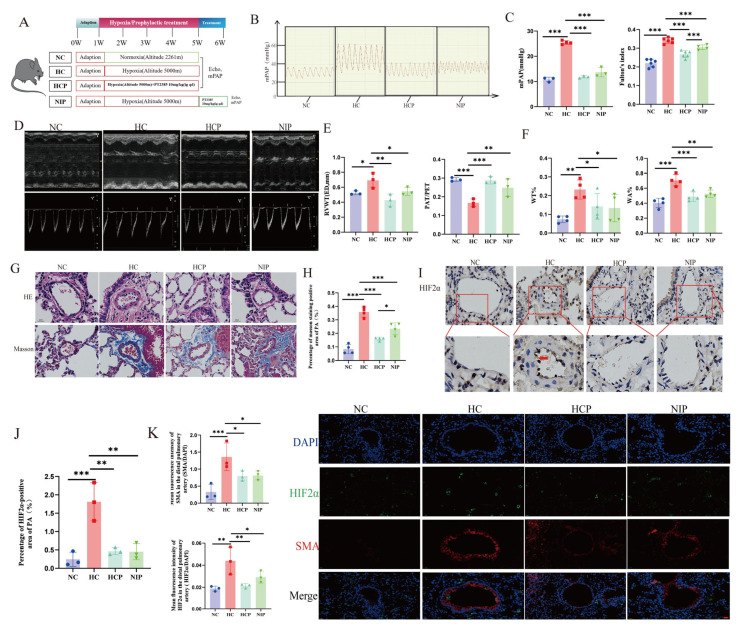
HIF2α inhibition attenuates chronic hypoxia-induced PH in vivo. (**A**) Graphical summary of animal modeling. (**B**) Images of the mPAP waves. (**C**) The summary data of mPAP and Fulton’s index. (**D**,**E**) Representative echocardiographic images and summary data of RVWT(ED) and PAT/PET. (**F**) The summary data from HE staining. (**G**) The representative images from HE staining and Masson staining of mice lung sections (scale bar, 20 μm, magnification, ×400). (**H**) The summary data from Masson staining. (**I**,**J**) The representative images and summary data from immuno-histochemical staining of mice pulmonary vasculature, red arrows in (**I**) indicate HIF2α-positive staining (scale bar, 20 μm, magnification, ×400). (**K**) The representative images and summary data from immunofluorescence of mice pulmonary vasculature, scale bars, 50 μm. NC: Normoxia control group; HC: Hypoxia control group; HCP: Hypoxia combined with PT2385 group (animal model); NIP: PT2385 intervention for 1 week after 4-week hypoxia exposure (animal model); mPAP: Mean pulmonary artery pressure; RVWT, ED: Right ventricular end-diastolic wall thickness; PAT/PET: Pulmonary artery acceleration time/pulmonary artery ejection time; WT%: Pulmonary artery wall thickness ratio; SMA: α-smooth muscle actin; In the statistical graph, each point represents the number of experimental animals.The data are presented as mean ± SD; * *p* < 0.05; ** *p* < 0.01; *** *p* < 0.0001.

To determine whether HIF2α inhibitors could block the development of HPH, we exposed mice to normoxic or hypoxic (10% O_2_) environments for 4 weeks. During the hypoxia exposure, PT2385 was administered as a prophylactic model. In addition, mice were kept in hypoxia for 4 weeks to build an HPH model, and then PT2385 was administered for 1 week as a treatment model (Figure 6A). The results showed that PT2385 attenuated hypoxia-triggered PH and right ventricular hypertrophy (Figure 6B,C); the data of echocardiography in mice also supported this conclusion (Figure 6D,E). The morphology analysis showed that PT2385 attenuated hypoxia-triggered PVR and fibrosis of the internal and external vascular membranes (Figure 6F–H). The inhibitory effect of PT2385 was also verified by immunohistochemistry and fluorescence (Figure 6I–K). 

**Figure 7:**
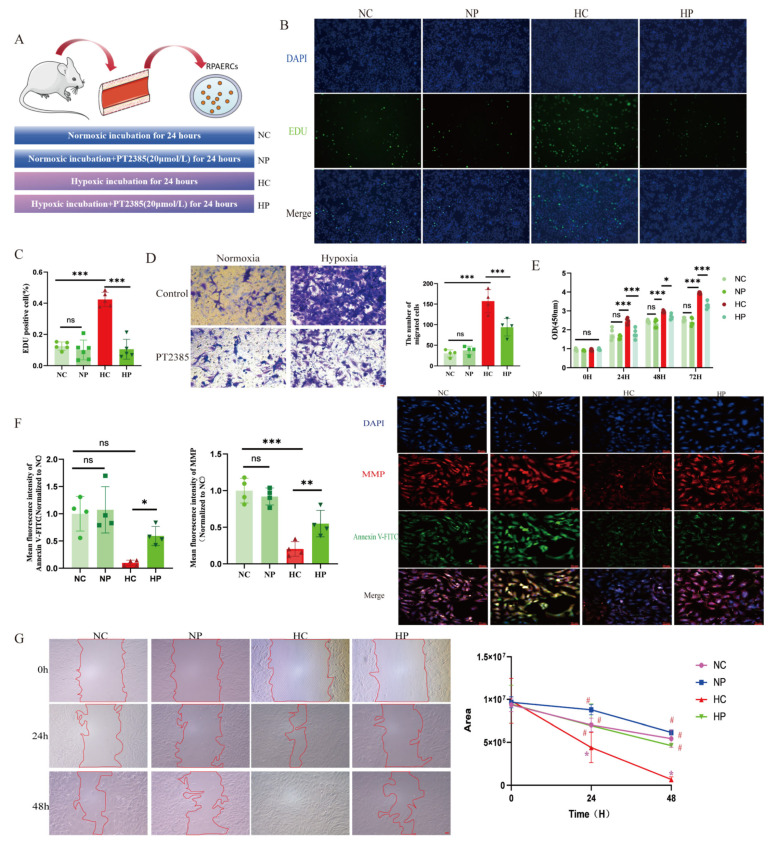
HIF2α inhibition improves chronic hypoxia-induced abnormal phenotypes of PAECs. (**A**) Graphical representation of detailed experimental methods for PT2385 intervention in RPAECs. (**B**,**C**) The typical images and summary of the EDU trial, scale bars = 50 μm. (**D**) The summary data and representative images of the Transwell assay in RPAECs, scale bars = 50 μm. (**E**) The summary data of the CCK8 assay. (**F**) The summary data and representative immunofluorescence images of MMP and Annexin V-FITC in RPAECs. (**G**) Wound healing assay of RPAECs in each group (* *p* < 0.05 vs. NC; # *p* < 0.05 vs. HC), scale bars, 50 μm. NC: Normoxia control group; NP: Normoxia combined with PT2385 group (cellular model); HC: Hypoxia control group; HP: Hypoxia combined with PT2385 group (cellular model); MMP: Mitochondrial membrane potential. In the statistical graph, each point represents the number of experimental repetitions. The data are presented as mean ± SD; ns: No statistical significance; * *p* < 0.05; ** *p* < 0.01; *** *p* < 0.0001.

In vitro experiments demonstrated that PT2385 did not affect RPAEC proliferation, migration, and apoptosis under normoxia. Chronic hypoxia, however, induced RPAEC hyper-proliferation, hypermigration, and apoptosis resistance, which were attenuated by PT2385 (Figure 7A–G). Furthermore, PT2385 partially restored hypoxia-induced mitochondrial membrane depolarization in RPAECs (Figure 7F).

### 3.6. HIF2α Inhibitor Reshapes the Metabolism of PAECs by Improving Mitochondrial Function

**Figure 8:**
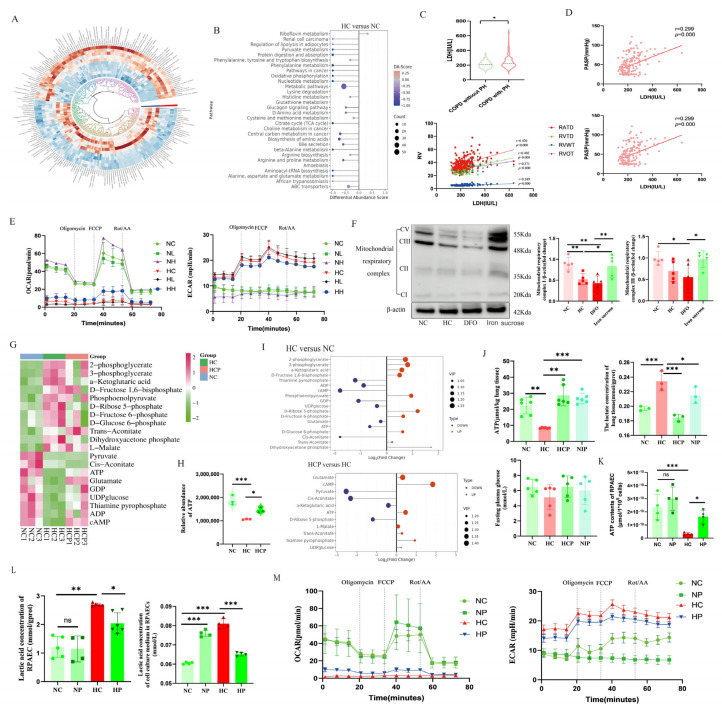
HIF2α inhibitor can reshape the energy metabolism of PAECs. (**A**) Non-targeted LC-MS-based metabolomics analysis of mice lung tissue. (**B**) Results of non-targeted metabolomics enrichment pathway analysis. (**C**,**D**) Serum LDH expression and correlation analysis in patients with chronic lung disease with or without HPH. (**E**) The OCAR and ECAR of RPAECs after iron intervention. (**F**) The expression levels of mitochondrial complexes in the lung tissues of rats. (**G**) Targeted energy metabolomics analysis of mouse lung tissue with PT2385 intervention. (**H**) Comparison of ATP abundance among groups in targeted metabolomics. (**I**) The metatolites analysis of targeted energy metabolomics. (**J**) The fasting blood glucose, lung tissue ATP, and lactate content in PT2385-intervened mice. (**K**,**L**) ATP and lactate content of RAPECs and culture medium. (**M**) The OCAR and ECAR of RPAECs after PT2385 intervention. NC: Normoxia control group; NP: Normoxia combined with PT2385 group; HC: Hypoxia control group; HP: Hypoxia combined with PT2385 group; NL: Normoxia combined with deferoxamine group; NH: Normoxia combined with iron sucrose group; HL: Hypoxia combined with deferoxamine group; HH: Hypoxia combined with iron sucrose group; DFO: Hypoxia combined with deferoxamine group; Iron sucrose: Hypoxia combined with iron sucrose group; HCP: Hypoxia combined with PT2385 group (animal model); LDH: Lactic dehydrogenase; OCAR: Oxygen consumption rate; ECAR: Extracellular acidification rate. The original images of Western Blot can be found in the Supplementary Files. In the statistical graph, each point represents the number of experimental animals or repetitions. The data are presented as mean ± SD; ns: No statistical significance; * *p* < 0.05; ** *p* < 0.01; *** *p* < 0.0001.

Metabolic reprogramming becomes one of the crucial mechanisms of PVR, which prompted us to detect metabolic changes in lung tissue in HPH and identify differential expression pathways. Non-targeted LC-MS metabolomics screening of lung tissue metabolites in normoxic and hypoxic control mice screened for 209 differential metabolites (Supplemental File: Figure S1L, Figure 8A), and metabolic pathway enrichment analyses showed that hypoxia down-regulated amino acid and energy metabolism, and that oxidative phosphorylation was markedly inhibited (Figure 8B). The results of the retrospective study of patients also supported this conclusion. The essential glycolytic enzyme LDH was higher among patients suffering from chronic hypoxic lung disease with HPH than those without HPH, and LDH was significantly positively correlated with PASP, right ventricular structure, and function index (Figure 8C,D). Further, we performed a Seahorse assay on RPAECs and found that iron supplementation corrected hypoxia-induced reduction in OCAR and elevation in ECAR (Figure 8E), and by Western blot assay, we found that iron supplementation improved the expression of mitochondrial complex I under hypoxia (Figure 8F). In brief, the data manifest that there is metabolic reprogramming of HPH and that iron supplementation can remodel PAECs’ metabolism by improving the activity of mitochondrial complex I under hypoxia.

To explore whether iron affects the development of HPH through HIF2α, we performed metabolomic assays targeting energy metabolism on lung tissues from a PT2385-intervened mouse model. The results showed that there were 18 discrepant metabolites between HC and NC groups (Figure 8G,I), and 11 differential metabolites between HCP and HC groups, among which the inhibition of ATP production induced by hypoxia was upregulated (Figure 8H). Further validation experiments showed that hypoxia induced decreases in fasting glucose in mice (Figure 8J), which is consistent with the previous finding of increased pulmonary vascular GLUT1 expression (Figure 2D,F), suggesting that pulmonary vascular cells consume more glucose under hypoxia, and indirectly indicating a metabolic mode of inefficient energy production in pulmonary vascular cells. Hypoxia significantly decreased ATP levels in both HPH mouse lung tissues and RPAECs, concomitant with elevated lactate concentrations (Figure 8J–L), and Seahorse results from PT2385-intervened RPAECs suggested that hypoxia caused a reduction in OCAR and an increase in ECAR (Figure 8M), and it is speculated that that PT2385 treatment may enhance endothelial ATP production by modulating the balance between oxidative phosphorylation and glycolysis (Figure 8J–M).

### 3.7. HIF2α Inhibitors Improve the Activity of Mitochondrial Complexes I and III, Reduce Mitochondrial ROS Production, and Attenuate Mitochondrial Respiratory Depression of PAECs Under Hypoxia 

**Figure 9:**
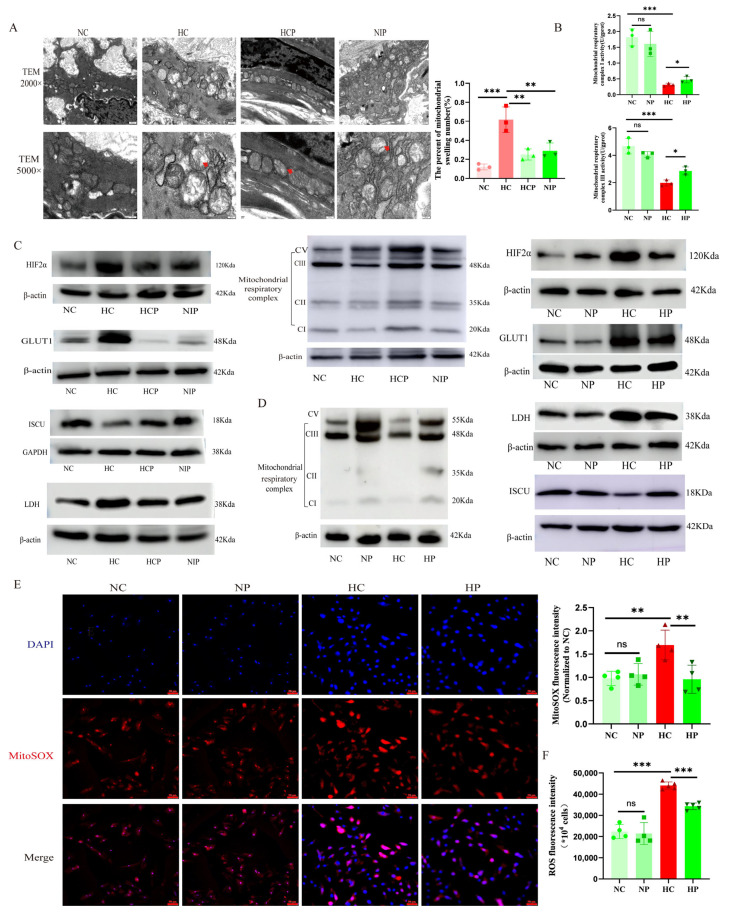
HIF2α inhibitors improve the activity and expression of mitochondrial complexes I and III, reduce mitochondrial ROS production, and attenuate mitochondrial respiratory depression of PAECs under hypoxia. (**A**) Representative transmission electron microscopy images of endothelial cells from mouse pulmonary arteries with PT2385 intervention (Red arrows indicate swollen mitochondria; scale bars: 500 nm (upper panel), 200 nm (lower panel). (**B**) Summary of data on the activity of mitochondrial complexes I and III in RPAECs. (**C**,**D**) The representative images of Western blot from mice lung tissues and RPAECs. (**E**) Representative immunofluorescence images and data of MitoSOX in RPAECs (scale bars: 50 μm). (**F**) Summary of data on the intracellular ROS. NC: Normoxia control group; NP: Normoxia combined with PT2385 group; HC: Hypoxia control group; HP: Hypoxia combined with PT2385 group; HCP: Hypoxia combined with PT2385 group (animal model); NIP: PT2385 intervention for 1 week after 4-week hypoxia exposure (animal model); LDH: Lactic dehydrogenase; GLUT1: Glucose transporter 1; ISCU: Iron/sulfur cluster assembly enzyme; MitoSOX: Mitochondrial superoxide; ROS: Reactive oxygen species. The original images of Western Blot can be found in the Supplementary Files. In the statistical graph, each point represents the number of experimental animals or repetitions. The data are presented as mean ± SD; ns: No statistical significance; * *p* < 0.05; ** *p* < 0.01; *** *p* < 0.0001.

Hypoxia significantly induced mitochondrial swelling in PAECs (Figure 9A), accompanied by upregulation of GLUT1 and LDH protein expression (Figure 9C,D and Figure S3A,B). Concurrently, hypoxia suppressed both enzymatic activity and protein levels of mitochondrial complexes I and III (Figure 9B–D). Mechanistically, chronic hypoxia downregulated the expression of ISCU, a critical cofactor for complexes I and III assembly (Figure 9C,D). Pharmacological inhibition of HIF2α with PT2385 significantly attenuated these hypoxia-induced abnormal alterations (Figure 9C,D).

Given that mitochondrial complexes I and III serve as primary sites for ROS generation under hypoxic conditions, we quantified both mitochondrial ROS and total intracellular ROS levels. PT2385 treatment effectively mitigated hypoxia-elevated ROS production, as evidenced by MitoSOX™ Red fluorescence assays (Figure 9E,F).

### 3.8. Targeting Mitochondrial ROS Relieves Mitochondrial Respiratory Depression and Improves the Dysfunction of PAECs Under Hypoxia

**Figure 10:**
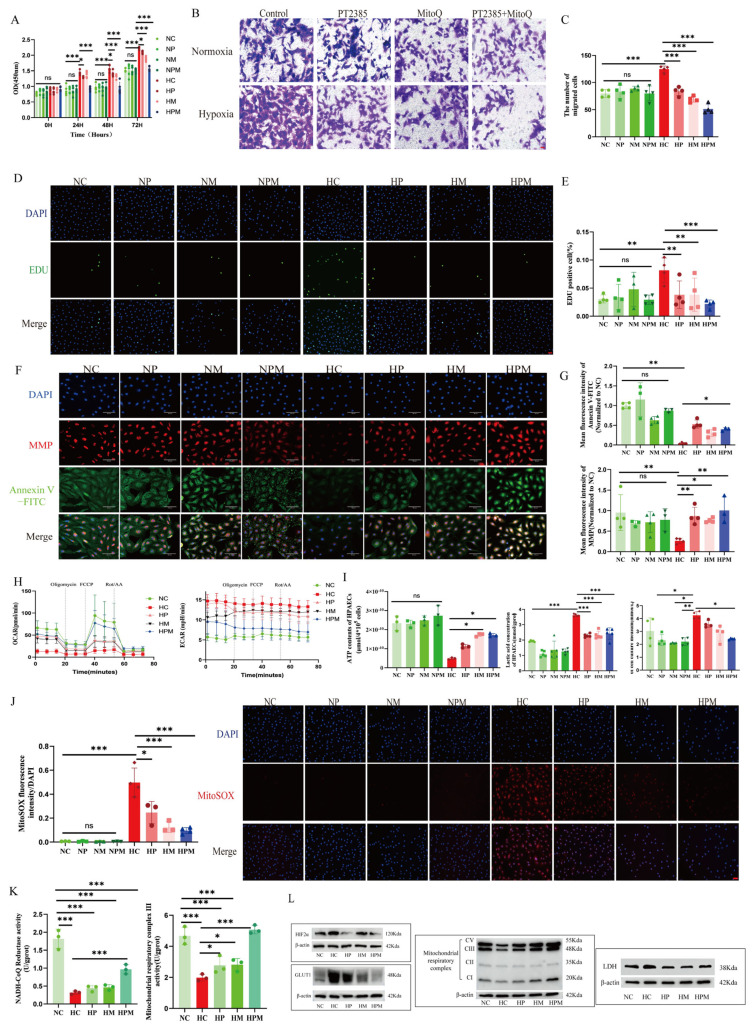
Targeting mitochondrial ROS relieves mitochondrial respiratory depression and improves the dysfunction of PAECs under hypoxia. (**A**) The summary data of the CCK8 assay in HPAECs. (**B**,**C**) Representative images and summary data of the Transwell assay, scale bars = 50 μm. (**D**,**E**) Representative images and summary data of EDU assay, scale bars = 50 μm. (**F**,**G**) Representative immunofluorescence images and summary data of MMP and Annexin V-FITC in HPAECs. (**H**) The summary data of OCAR and ECAR in HPAECs. (**I**) The summary data of ATP, lactic acid concentration of HPAECs. (**J**) Representative immunofluorescence images and summary data of MitoSOX in HPAECs, scale bars = 50 μm. (**K**) Summary of data on the activity of mitochondrial complexes I and III in HPAECs. (**L**) The representative images of target proteins in HPEACs from Western blot analysis. NC: Normoxia control group; NP: Normoxia combined with PT2385 group; NM: Normoxia combined with MitoQ group; NPM: Normoxia combined with PT2385 and MitoQ group; HC: Hypoxia control group; HP: Hypoxia combined with PT2385 group; HM: Hypoxia combined with MitoQ group; HPM: Hypoxia combined with PT2385 and MitoQ group; MMP: Mitochondrial membrane potential; OCAR: oxygen consumption rate; ECAR: Extracellular acidification rate; MitoSOX: Mitochondrial superoxide; ROS: Reactive oxygen species. The original images of Western Blot can be found in the Supplementary Files. In the statistical graph, each point represents the number of experimental repetitions.The data are presented as mean ± SD; ns: No statistical significance; * *p* < 0.05; ** *p* < 0.01; *** *p* < 0.0001.

To further investigate whether ROS may be a prospective treatment target for HPH, we evaluated the role of the mitochondrial ROS scavenger MitoQ in HPAECs (Figure S2A). We found that MitoQ attenuated hypoxia-induced hyper-proliferation, migration, and anti-apoptotic phenotypes in HPAECs (Figure 10A–G) by restoring the oxidative phosphorylation/glycolysis balance via enhanced ATP production and reduced lactate accumulation (Figure 10H,I). Mechanistically, MitoQ improved the activity and expression of mitochondrial complexes I, III, and IV under hypoxia (Figure 10K,L and Figure S2B), concomitant with diminished mitochondrial ROS generation (Figure 10J). These effects were potentiated by co-treatment with PT2385. It should be particularly noted that the expression of HIF2α was not affected by MitoQ intervention (Figure 10L and Figure S2B), and we speculate that HIF2α may unidirectionally regulate mitochondrial ROS yield. Overall, the data illustrate that targeting mitochondrial ROS relieves mitochondrial respiration depression and ameliorates the dysfunction of PAECs under hypoxia.

## 4. Discussion

Plateau HPH is a severe and fatal disease. To date, there are no effective drugs for the treatment of plateau HPH [39]. Therefore, screening for new targets of intervention may be a potential approach to treat HPH. Notably, PVR is a crucial pathogenic alteration in HPH, and iron homeostatic imbalance is a key player in initiating and developing such a process. Recently, some clinical studies have indicated that iron deficiencies are widespread among patients suffering from group 1 PH and connected to a remarkable decrease in exercise capacity, a significant deterioration of clinical symptoms, and a decline in survival of patients with PH [11,12,13,14]. Here, we observed that chronic hypoxia leads to disorders of iron metabolism. Patients with HPH caused by chronic lung diseases at plateau typically exhibited decreased serum iron levels and TSAT saturation. Meanwhile, the animal model of HPH mainly showed reduced serum iron and ferritin levels. These observations are consistent with previous research findings [15,35]. Disturbed iron metabolism may be related to the expression of Hepcidin, a significant factor that regulates iron metabolism, and in our study, we found that hypoxia induced an elevation of hepatic Hepcidin, which binds to ferroportin, the only cellular iron export factor, and inhibits intracellular iron output. The dysregulation of hepatic Hepcidin expression may be related to the BMPR/SMD pathway, an essential regulator of Hepcidin expression [35]. Similarly, PH due to iron deficiency has been studied, with results from animal experiments suggesting that chronic iron deficiency diets lead to PVR under normoxia and that iron replacement attenuates low iron-induced PVR [15]. In patients with chronic high-altitude diseases, iron deficiency caused by phlebotomy can also exacerbate pulmonary hypertension [17]. Intriguingly, it is unclear whether low iron exerts a dual pathogenic role under chronic hypoxia. Moreover, our results indicated that hypoxia combined with iron deficiency aggravated PH and PVR. In contrast, iron supplementation attenuated hypoxia-induced PH and PVR. This provides important references for the function of iron supplementation in HPH.

Furthermore, our discoveries demonstrate that iron deficiencies are associated with the activation of HIF2α and that, as proline hydroxylase requires iron as a cofactor, the underlying mechanism may involve hypoxic stabilization of HIF2α. HIF2α, a critical transcription factor for hypoxic adaptation, is abundantly expressed mainly in endothelial cells, a finding confirmed by our immunofluorescence results. In addition, previous studies have confirmed that HIF2α inhibitors or endothelium-specific deletion of HIF2α improve PVR and attenuate PH [40,41,42,43,44,45]. Different from these studies, the HIF2α inhibitor we chose was PT2385, which has been used in a phase II clinical trial for treating renal clear cell carcinoma and has been shown to be safer and more efficacious than other HIF2α inhibitors in humans [46,47]. Our findings further demonstrate that PT2385 significantly attenuates hypoxia-induced PH and PVR. These results provide compelling preclinical evidence supporting the therapeutic potential of HIF2α inhibitors in HPH. 

Previous studies show that HIFs play a crucial role in regulating the metabolic shift in embryonic fibroblasts and nerve cells. The activation of HIF2α inhibits mitochondrial oxidative phosphorylation. The administration of HIF2α inhibitors can regulate mitochondrial morphology and quality and reverse the energy metabolic shift from glycolysis to oxidative phosphorylation [24,25,48]. The precise mechanisms by which HIF2α impairs mitochondrial function remain incompletely characterized. Emerging evidence suggests that HIF2α activation downregulates iron/sulfur cluster assembly factors (e.g., ISCU and frataxin), which are essential for the biogenesis of mitochondrial complexes I–III. These cofactors mediate electron transport through iron/sulfur clusters, and their deficiency may directly contribute to respiratory chain dysfunction under hypoxic conditions [20,24,49,50]. Our research results show that HIF2α inhibitors can reverse the reduction in ISCU expression induced by chronic hypoxia, thereby restoring the activities and protein content of mitochondrial complexes I and III under hypoxia. Notably, mitochondrial iron/sulfur clusters are integral components of complexes I–III, which serve as primary sites for physiological ROS generation. Under hypoxic conditions, complexes I and III remain major ROS-producing sites [20,21,51]. This functional interplay mechanistically links HIF-2α signaling, iron/sulfur cluster biogenesis, and ROS homeostasis, suggesting a hypoxia-adaptive pathway where HIF2α modulates mitochondrial redox balance via iron/sulfur-dependent electron transport regulation.

Accumulating evidence indicates that mitochondrial ROS play a crucial role in signaling the mismatch between energy production and demand. Given our finding that HIF2α inhibitors alleviate hypoxia-induced suppression of mitochondrial complexes I and III, we investigated mitochondrial ROS alterations under hypoxia. We discovered that mitochondrial ROS production was elevated in PAECs under hypoxia, and that PT2385 inhibited the hypoxia-induced augmentation of mitochondrial ROS production. MitoQ, a targeted mitochondrial ROS scavenger, has been used to treat pulmonary artery smooth muscle cells and cardiomyocytes exposed to chronic hypoxia. However, it was found to be ineffective in hypoxia-induced phenotypic alterations in the above-described smooth muscle cells, but effective in cardiomyocytes [52]. We speculate that this may be determined by the cell type, and future validation of animal models of endothelial cell-specific clearance of mitochondrial ROS is needed.

This study has several limitations that are worth considering. Firstly, while genetic knockout models could provide deeper mechanistic insights, our experimental design prioritized pharmacological interventions with PT2385—a clinically validated HIF2α inhibitor currently in phase II trials for renal carcinoma. Given its established safety profile and translational potential for repurposing in HPH, we focused on therapeutic-relevant approaches rather than exploratory genetic manipulations. Secondly, human lung tissue analyses were precluded by limited access to pulmonary vascular specimens from HPH patients. Future studies should incorporate precision-cut lung slices or patient-derived organoid models to bridge this gap.

## 5. Conclusions

In summary, we found for the first time that low iron combined with hypoxia activated HIF2α activity of PAECs, which aggravated the progression of HPH, and iron supplementation attenuated hypoxia-induced HPH. In addition, HIF2α inhibition improved PAECs dysfunction and attenuated hypoxia-triggered PH and PVR by relieving hypoxia-induced mitochondrial complexes I and III depression, reducing mitochondrial ROS production, and prompting the conversion of energy metabolism from glycolysis to oxidative phosphorylation. The data indicate that appropriate iron supplementation might be a potential strategy in the treatment of HPH, and that therapies targeting energy metabolism by inhibiting HIF2α activation may also be beneficial if iron deficiency status persists or fails to improve. Moreover, mitochondrial ROS of PAECs may also be a potential target for HPH therapy. The synergistic combination of these targets holds significant promise for clinical translation, presenting high clinical value. It is anticipated that these therapeutic approaches have the potential to fundamentally prevent, treat, and even reverse HPH.

## Data Availability

All data are included in the article and Supplementary File.

## Supplementary Materials

The following supporting information can be downloaded at: https://www.mdpi.com/article/10.3390/biom15050742/s1, Table S1: OPLS-DA model evaluation parameters for comparison group of non-targeted metabolomics; Table S2: A summary of the typical precursor-product ion-pairs of targeted energy metabolites; Table S3: OPLS-DA model evaluation parameters for each comparison group of targeted energy metabolomics; Table S4: Baseline features of chronic hypoxic lung disease patients with or without PH; Figure S1: The metabolomics datasets, cellular validation results, and retrospective clinical patient data analyses; Figure S2: The detailed grouping diagram of HPAECs experiments and quantitative data of Western blot analysis for pharmacological intervention in HPAECs. Figure S3: The quantitative results of Western blot analysis for PT2385 intervention in mouse lung tissues and RPAECs; Supplementary File: The original images of Western Blot.

## Abbreviations

HPH	Hypoxic pulmonary hypertension
PH	Pulmonary hypertension
COPD	Chronic obstructive pulmonary disease
PAECs	Pulmonary artery endothelial cells
RPAECs	Rat pulmonary artery endothelial cells
HPAECs	Human pulmonary artery endothelial cells
ROS	Reactive oxygen species
HIF2α	Hypoxia-inducible factor 2α
HIF1α	Hypoxia-inducible factor 1α
mPAP	Mean pulmonary artery pressure
IRP2	Iron regulatory protein 2
PAT	Pulmonary acceleration time
PET	Pulmonary ejection time
RVOT	Right ventricular outflow tract
RVWT, ED	Right ventricular end-diastolic wall thickness
RVWT, SD	Right ventricular end-systolic wall thickness
RV	Right ventricle
LV	Left ventricle
S	Septum
H&E	Hematoxylin and Eosin staining
WT %	Pulmonary artery wall thickness ratio
WA %	Pulmonary artery wall areas ratio
GLUT1	Glucose transporter 1
SMA	Smooth muscle actin
LDH	Lactate dehydrogenase
GAPDH	Glyceraldehyde-3-Phosphate Dehydrogenase
FC	Fold change
MitoQ	Mitoquinone mesylate
OCR	Oxygen consumption rate
ECAR	Extracellular acidification rate
FCCP	Trifluoromethoxy carbonyl cyanide phenylhydrazone
TSAT	Transferrin saturation
PASP	Pulmonary arterial systolic pressure
PHD	Prolyl hydroxylase
NO	Nitric oxide
MMP	Mitochondrial membrane potential
BMPR/SMD	Bone morphogenetic protein receptor/mothers against decapentaplegic homolog

## References

[1] Mocumbi A., Humbert M., Saxena A., Jing Z.C., Sliwa K., Thienemann F., Archer S.L., Stewart S. (2024). Pulmonary hypertension. Nat. Rev. Dis. Primers.

[2] Rose L., Prins K.W., Archer S.L., Pritzker M., Weir E.K., Misialek J.R., Thenappan T. (2019). Survival in pulmonary hypertension due to chronic lung disease: Inflfluence of low diffusion capacity of the lungs for carbon monoxide. J. Heart Lung Transpl. Transplant..

[3] Olsson K.M., Corte T.J., Kamp J.C., Montani D., Nathan S.D., Neubert L., Price L.C., Kiely D.G. (2023). Pulmonary hypertension associated with lung disease: New insights into pathomechanisms, diagnosis, and management. Lancet Respir. Med..

[4] Galiè N., Humbert M., Vachiery J.L., Gibbs S., Lang I., Torbicki A., Simonneau G., Peacock A., Vonk Noordegraaf A., Beghetti M. (2015). 2015 ESC/ERS Guidelines for the diagnosis and treatment of pulmonary hypertension: The Joint Task Force for the Diagnosis and Treatment of Pulmonary Hypertension of the European Society of Cardiology (ESC) and the European Respiratory Society (ERS): Endorsed by: Association for European Paediatric and Congenital Cardiology (AEPC), International Society for Heart and Lung Transplantation (ISHLT). Eur. Respir. J..

[5] Stenmark K.R., Meyrick B., Galie N., Mooi W.J., McMurtry I.F. (2009). Animal models of pulmonary arterial hypertension: The hope for etiological discovery and pharmacological cure. Am. J. Physiol. Lung Cell Mol. Physiol..

[6] Pokharel M.D., Marciano D.P., Fu P., Franco M.C., Unwalla H., Tieu K., Fineman J.R., Wang T., Black S.M. (2023). Metabolic reprogramming, oxidative stress, and pulmonary hypertension. Redox Biol..

[7] Caruso P., Dunmore B.J., Schlosser K., Schoors S., Dos Santos C., Perez-Iratxeta C., Lavoie J.R., Zhang H., Long L., Flockton A.R. (2017). Identification of MicroRNA-124 as a Major Regulator of Enhanced Endothelial Cell Glycolysis in Pulmonary Arterial Hypertension via PTBP1 (Polypyrimidine Tract Binding Protein) and Pyruvate Kinase M2. Circulation.

[8] Cuthbertson I., Morrell N.W., Caruso P. (2023). BMPR2Mutation and Metabolic Reprogramming in Pulmonary Arterial Hypertension. Circ. Res..

[9] Lu G.F., Geng F., Deng L.P., Lin D.C., Huang Y.Z., Lai S.M., Lin Y.C., Gui L.X., Sham J.S.K., Lin M.J. (2022). Reduced CircSMOC1 Level Promotes Metabolic Reprogramming via PTBP1 (Polypyrimidine Tract-Binding Protein) and miR-329-3p in Pulmonary Arterial Hypertension Rats. Hypertension.

[10] Liu D., Qin S., Su D., Wang K., Huang Y., Huang Y., Pang Y. (2021). Metabolic Reprogramming of the Right Ventricle and Pulmonary Arteries in a Flow-Associated Pulmonary Arterial Hypertension Rat Model. ACS Omega.

[11] Martens P., Yu S., Larive B., Borlaug B.A., Erzurum S.C., Farha S., Finet J.E., Grunig G., Hemnes A.R., Hill N.S. (2023). Iron deficiency in pulmonary vascular disease: Pathophysiological and clinical implications. Eur. Heart J..

[12] Kramer T., Wissmüller M., Natsina K., Gerhardt F., Ten Freyhaus H., Dumitrescu D., Viethen T., Hellmich M., Baldus S., Rosenkranz S. (2021). Ferric carboxymaltose in patients with pulmonary arterial hypertension and iron deficiency: A long-term study. J. Cachexia Sarcopenia Muscle.

[13] Soon E., Treacy C.M., Toshner M.R., MacKenzie-Ross R., Manglam V., Busbridge M., Sinclair-McGarvie M., Arnold J., Sheares K.K., Morrell N.W. (2011). Unexplained iron deficiency in idiopathic and heritable pulmonary arterial hypertension. Thorax.

[14] Ruiter G., Lankhorst S., Boonstra A., Postmus P.E., Zweegman S., Westerhof N., van der Laarse W.J., Vonk-Noordegraaf A. (2011). Iron deficiency is common in idiopathic pulmonary arterial hypertension. Eur. Respir. J..

[15] Cotroneo E., Ashek A., Wang L., Wharton J., Dubois O., Bozorgi S., Busbridge M., Alavian K.N., Wilkins M.R., Zhao L. (2015). Iron homeostasis and pulmonary hypertension: Iron deficiency leads to pulmonary vascular remodeling in the rat. Circ. Res..

[16] Naito Y., Hosokawa M., Hao H., Sawada H., Hirotani S., Iwasaku T., Okuhara Y., Eguchi A., Hirota S., Ohyanagi M. (2013). Impact of dietary iron restriction on the development of monocrotaline-induced pulmonary vascular remodeling and right ventricular failure in rats. Biochem. Biophys. Res. Commun..

[17] Smith T.G., Talbot N.P., Privat C., Rivera-Ch M., Nickol A.H., Ratcliffe P.J., Dorrington K.L., León-Velarde F., Robbins P.A. (2009). Effects of iron supplementation and depletion on hypoxic pulmonary hypertension: Two randomized controlled trials. JAMA.

[18] Sommer N., Pak O., Schörner S., Derfuss T., Krug A., Gnaiger E., Ghofrani H.A., Schermuly R.T., Huckstorf C., Seeger W. (2010). Mitochondrial cytochrome redox states and respiration in acute pulmonary oxygen sensing. Eur. Respir. J..

[19] Siques P., Brito J., Pena E. (2018). Reactive Oxygen Species and Pulmonary Vasculature During Hypobaric Hypoxia. Front. Physiol..

[20] Guzy R.D., Hoyos B., Robin E., Chen H., Liu L., Mansfield K.D., Simon M.C., Hammerling U., Schumacker P.T. (2005). Mitochondrial complex III is required for hypoxia-induced ROS production and cellular oxygen sensing. Cell Metab..

[21] Okoye C.N., Koren S.A., Wojtovich A.P. (2023). Mitochondrial complex I ROS production and redox signaling in hypoxia. Redox Biol..

[22] Uchida T., Rossignol F., Matthay M.A., Mounier R., Couette S., Clottes E., Clerici C. (2004). Prolonged hypoxia differentially regulates hypoxia-inducible factor (HIF)-1alpha and HIF-2alpha expression in lung epithelial cells: Implication of natural antisense HIF-1alpha. J. Biol. Chem..

[23] Metzen E., Ratcliffe P.J. (2004). HIF hydroxylation and cellular oxygen sensing. Biol. Chem..

[24] Li H., Liu Y., Shang L., Cai J., Wu J., Zhang W., Pu X., Dong W., Qiao T., Li K. (2019). Iron regulatory protein 2 modulates the switch from aerobic glycolysis to oxidative phosphorylation in mouse embryonic fibroblasts. Proc. Natl. Acad. Sci. USA.

[25] Shen J., Xu L., Li Y., Dong W., Cai J., Liu Y., Zhao H., Xu T., Holtz E.M., Chang Y. (2021). Protective Effects of Hif2 Inhibitor PT-2385 on a Neurological Disorder Induced by Deficiency of Irp2. Front. Neurosci..

[26] Yao C., Weng J., Feng L., Zhang W., Xu Y., Zhang P., Tanaka Y., Su L. (2022). SIPA1 Enhances Aerobic Glycolysis Through HIF-2α Pathway to Promote Breast Cancer Metastasis. Front. Cell Dev. Biol..

[27] Sanchez M., Galy B., Muckenthaler M.U., Hentze M.W. (2007). Iron-regulatory proteins limit hypoxia-inducible factor-2alpha expression in iron deficiency. Nat. Struct. Mol. Biol..

[28] Luo Y., Qi X., Zhang Z., Zhang J., Li B., Shu T., Li X., Hu H., Li J., Tang Q. (2024). Inactivation of Malic Enzyme 1 in Endothelial Cells Alleviates Pulmonary Hypertension. Circulation.

[29] Ji L., Su S., Xin M., Zhang Z., Nan X., Li Z., Lu D. (2022). Luteolin ameliorates hypoxia-induced pulmonary hypertension via regulating HIF-2α-Arg-NO axis and PI3K-AKT-eNOS-NO signaling pathway. Phytomedicine.

[30] Dongiovanni P., Valenti L., Ludovica Fracanzani A., Gatti S., Cairo G., Fargion S. (2008). Iron depletion by deferoxamine upregulates glucose uptake and insulin signaling in hepatoma cells and in rat liver. Am. J. Pathol..

[31] Xie C., Yagai T., Luo Y., Liang X., Chen T., Wang Q., Sun D., Zhao J., Ramakrishnan S.K., Sun L. (2017). Activation of intestinal hypoxia-inducible factor 2α during obesity contributes to hepatic steatosis. Nat. Med..

[32] Zhu Z., Godana D., Li A., Rodriguez B., Gu C., Tang H., Minshall R.D., Huang W., Chen J. (2019). Echocardiographic assessment of right ventricular function in experimental pulmonary hypertension. Pulm. Circ..

[33] Frump A.L., Selej M., Wood J.A., Albrecht M., Yakubov B., Petrache I., Lahm T. (2018). Hypoxia Upregulates Estrogen Receptor β in Pulmonary Artery Endothelial Cells in a HIF-1α-Dependent Manner. Am. J. Respir. Cell Mol. Biol..

[34] Shi Y., Liu J., Zhang R., Zhang M., Cui H., Wang L., Cui Y., Wang W., Sun Y., Wang C. (2023). Targeting Endothelial ENO1 (Alpha-Enolase) -PI3K-Akt-mTOR Axis Alleviates Hypoxic Pulmonary Hypertension. Hypertension.

[35] Jiang Y., Guo Y., Feng X., Yang P., Liu Y., Dai X., Zhao F., Lei D., Li X., Liu Y. (2023). Iron metabolism disorder regulated by BMP signaling in hypoxic pulmonary hypertension. Biochim. Biophys. Acta Mol. Basis Dis..

[36] Hsu T.S., Lin Y.L., Wang Y.A., Mo S.T., Chi P.Y., Lai A.C., Pan H.Y., Chang Y.J., Lai M.Z. (2020). HIF-2α is indispensable for regulatory T cell function. Nat. Commun..

[37] Mitchell T., Rotaru D., Saba H., Smith R.A., Murphy M.P., MacMillan-Crow L.A. (2011). The mitochondria-targeted antioxidant mitoquinone protects against cold storage injury of renal tubular cells and rat kidneys. J. Pharmacol. Exp. Ther..

[38] Lan M., Wu S., Fernandes T.M. (2022). Iron deficiency and pulmonary arterial hypertension. Nutr. Clin. Pract..

[39] Naeije R. (2019). Pulmonary hypertension at high altitude. Eur. Respir. J..

[40] Hu C.J., Poth J.M., Zhang H., Flockton A., Laux A., Kumar S., McKeon B., Mouradian G., Li M., Riddle S. (2019). Suppression of HIF2 signaling attenuates the initiation of hypoxia-induced pulmonary hypertension. Eur. Respir. J..

[41] Dai Z., Zhu M.M., Peng Y., Machireddy N., Evans C.E., Machado R., Zhang X., Zhao Y.Y. (2018). Therapeutic Targeting of Vascular Remodeling and Right Heart Failure in Pulmonary Arterial Hypertension with a HIF-2α Inhibitor. Am. J. Respir. Crit. Care Med..

[42] Ghosh M.C., Zhang D.L., Ollivierre W.H., Noguchi A., Springer D.A., Linehan W.M., Rouault T.A. (2021). Therapeutic inhibition of HIF-2α reverses polycythemia and pulmonary hypertension in murine models of human diseases. Blood.

[43] Macias D., Moore S., Crosby A., Southwood M., Du X., Tan H., Xie S., Vassallo A., Wood A.J.T., Wallace E.M. (2021). Targeting HIF2α-ARNT hetero-dimerisation as a novel therapeutic strategy for pulmonary arterial hypertension. Eur. Respir. J..

[44] Cowburn A.S., Crosby A., Macias D., Branco C., Colaço R.D., Southwood M., Toshner M., Crotty Alexander L.E., Morrell N.W., Chilvers E.R. (2016). HIF2α-arginase axis is essential for the development of pulmonary hypertension. Proc. Natl. Acad. Sci. USA.

[45] Brusselmans K., Compernolle V., Tjwa M., Wiesener M.S., Maxwell P.H., Collen D., Carmeliet P. (2003). Heterozygous deficiency of hypoxia-inducible factor-2alpha protects mice against pulmonary hypertension and right ventricular dysfunction during prolonged hypoxia. J. Clin. Investig..

[46] Courtney K.D., Infante J.R., Lam E.T., Figlin R.A., Rini B.I., Brugarolas J., Zojwalla N.J., Lowe A.M., Wang K., Wallace E.M. (2018). Phase I Dose-Escalation Trial of PT2385, a First-in-Class Hypoxia-Inducible Factor-2α Antagonist in Patients with Previously Treated Advanced Clear Cell Renal Cell Carcinoma. J. Clin. Oncol..

[47] Courtney K.D., Ma Y., Diaz de Leon A., Christie A., Xie Z., Woolford L., Singla N., Joyce A., Hill H., Madhuranthakam A.J. (2020). HIF-2 Complex Dissociation, Target Inhibition, and Acquired Resistance with PT2385, a First-in-Class HIF-2 Inhibitor, in Patients with Clear Cell Renal Cell Carcinoma. Clin. Cancer Res..

[48] Almasi S., SarmastiEmami S., Baird S., Ning Z., Figeys D., Côté J., Cowan K.N., Jasmin B.J. (2023). Staufen1 controls mitochondrial metabolism via HIF2α in embryonal rhabdomyosarcoma and promotes tumorigenesis. Cell Mol. Life Sci..

[49] Oktay Y., Dioum E., Matsuzaki S., Ding K., Yan L.J., Haller R.G., Szweda L.I., Garcia J.A. (2007). Hypoxia-inducible factor 2alpha regulates expression of the mitochondrial aconitase chaperone protein frataxin. J. Biol. Chem..

[50] Hervouet E., Cízková A., Demont J., Vojtísková A., Pecina P., Franssen-van Hal N.L., Keijer J., Simonnet H., Ivánek R., Kmoch S. (2008). HIF and reactive oxygen species regulate oxidative phosphorylation in cancer. Carcinogenesis.

[51] Guzy R.D., Mack M.M., Schumacker P.T. (2007). Mitochondrial complex III is required for hypoxia-induced ROS production and gene transcription in yeast. Antioxid. Redox Signal..

[52] Pak O., Scheibe S., Esfandiary A., Gierhardt M., Sydykov A., Logan A., Fysikopoulos A., Veit F., Hecker M., Kroschel F. (2018). Impact of the mitochondria-targeted antioxidant MitoQ on hypoxia-induced pulmonary hypertension. Eur. Respir. J..

