# Supplementary material for: Iron Replacement Attenuates Hypoxic Pulmonary Hypertension by Remodeling Energy Metabolism via Regulating the HIF2α/Mitochondrial Complex I, III/ROS Axis
Source: Biomolecules. 2025 May 21;15(5):742. doi: 10.3390/biom15050742 (PMC12109292; doi:10.3390/biom15050742)
Supplement: Supplementary file 1 [file biomolecules-15-00742-s001.zip › biomolecules-3579377-supplementary.pdf]

Table.S1. OPLS-DA model evaluation parameters for comparison group of non-targeted metabolomics

**Sample Comparison Groups**	**R2X(cum)**	**R2Y(cum)**	**Q2(cum)**	**RMSEE**	**pre**	**ort**	**pR2Y**	**pQ2**
HC_NC	0.368	0.997	0.815	0.0326	1	1	0.025	0.015

Note: NC: Normoxia control group; HC: Hormoxia control group; R2X and R2Y denote the explanation rate of the model on the X and Y matrices; Q2: denotes the model prediction ability; the closer R2 and Q2 are to 1 indicates that the model is more stable and reliable, Q2 > 0.5 indicates that the model's prediction ability is better, and Q2 less than 0.5 indicates that the model's prediction ability is poorer; RMSEE: root mean square error of the estimation; pre denotes the prediction group scores used for modelling, ort denotes the orthogonal group scores used for modelling; pR2Y: the p-value of R2Y, pQ2: the *p*-value of R2Y orthogonal group scores for modelling; pR2Y:*p*-value of R2Y, pQ2:*p*-value of Q2.

Table.S2. A summary of the typical precursor-product ion-pairs of targeted energy metabolites

Component Name	KEGG	ESI Mode	RT(min)	Transition	QC1	QC2	QC3	QCRSD
2-phosphoglycerate	C00631	Negative	11.42	185/79	37090025.96	31851896.23	31928825.54	8.93%
3-phosphoglycerate	C00197	Negative	11.38	185/97	38219504.04	38892004.72	39709213.04	1.92%
6-phosphogluconate	C00345	Negative	11.58	275/97	4716946	4569837.505	4635169.835	1.59%
Acetyl-CoA	C00024	Negative	10.95	808/408	17196.82665	17967.6129	19977.74608	7.81%
ADP	C00008	Negative	11.45	426/134	22157234.48	23094964.84	23362647.12	2.77%
ADPglucose	C00498	Negative	11.08	588/79	767052.3698	809949.6528	775039.6207	2.91%
alpha-Ketoglutarate	C00026	Negative	9.94	145/101	44493352.68	47715092.96	45614494.35	3.56%
AMP	C00020	Negative	10.88	346/79	114274825.4	118026190.4	114177353.6	1.90%
ATP	C00002	Negative	11.51	506/159	1345045.888	1397869.962	1179719.253	8.70%
cAMP	C00575	Negative	8	328/134	406213.5648	411744.3247	422780.4971	2.04%
Cis-Aconitate	C00417	Negative	10.29	173/129	2212366.804	2563528.764	2393852.458	7.35%
Citrate	C00158	Negative	11.65	191/111	368954290.7	360423625.3	437046867.6	10.80%
D-Fructose 1,6-bisphosphate	C00354	Negative	11.51	339/79	49612814.94	47656620.72	46679539.51	3.11%
D-Fructose 6-phosphate	C00085	Negative	11.33	259/79	107690801.3	100593892.6	99500627.21	4.33%

D-Glucose 1-phosphate	C00103	Negative	11.46	259/241	4151618.411	4197904.214	3872566.185	4.32%
D-Glucose 6-phosphate	C00092	Negative	11.32	259/79.001	104295775.3	97517860.4	101503696.7	3.37%
Dihydroxyacetone phosphate	C00111	Negative	11.29	169/97.01	3142451.182	4088413.083	4285186.198	15.92%
D-Ribose 5-phosphate	C00117	Negative	11.18	229/97	40605692.78	44121941.54	43442320.74	4.37%
Fumarate	C00122	Negative	9.89	115/71	29813889.83	36355940.38	35818210.55	10.68%
GDP	C00035	Negative	11.51	442/79	1504607.605	1488138.978	1575933.337	3.06%
Glyceraldehyde 3-phosphate	C00661	Negative	11.28	169/97	3563377.294	4061271.502	4123373.913	7.84%
GTP	C00044	Negative	11.59	522/79	233077.5137	238490.892	237029.2591	1.19%
Isocitrate	C00311	Negative	11.43	191/73	6591919.582	6051756.159	5926918.256	5.71%
L-Glutamate	C00025	Negative	10.17	146/128	144431809.1	147465671.3	146771359.9	1.09%
L-Lactate	C00186	Negative	5.11	89/45	216477085.1	231447683.9	233640413.7	4.11%
L-Malate	C00149	Negative	11.38	133/115	258273048.9	247664441.5	234598534.6	4.80%
NAD	C00003	Positive	11.05	664.1/136.3	934609.596	1187798.635	1121873.473	12.15%
NADH	C00004	Negative	11.46	664/79	720986.6434	771171.1739	858238.2899	8.86%
NADP	C00006	Negative	11.45	742/620	134445.9435	122224.7356	115115.9244	7.89%
NADPH	C00005	Negative	11.45	744/408	19463.32119	13804.06066	17264.21984	16.94%
Oxaloacetate	C00036	Negative	5.28	131/87	4974321.886	4656754.452	5626560.903	9.72%

Phosphoenolpyruvate	C00074	Negative	11.38	167/79	15443591.8	14436316.32	15369992.69	3.72%
Pyruvate	C00022	Negative	2.57	87/43	7292009.789	7349685.294	7362568.23	0.51%
Ribulose-1, 5 bisphosphate	C01182	Negative	11.51	309/79	1015194.001	984231.0246	979894.4532	1.94%
Succinate	C00042	Negative	9.7	117/73	84055188.3	81682945.86	81092070.82	1.91%
Succinyl-CoA	C00091	Positive	11.35	868.1/361	3193.194175	3962.798454	3909.454541	11.65%
Thiamine pyrophosphate	C00068	Negative	11.55	423.1/302	779036.4048	796620.5321	740689.731	3.70%
Trans-Aconitate	C02341	Negative	11.18	173/85.001	37088765.15	38293261.38	38432605.69	1.95%
UDPglucose	C00029	Negative	11.19	565/323	50688690.54	50162793.43	51345318.14	1.17%
FMN	C00061	Negative	10.46	455/213	N/A	N/A	N/A	#DIV/0!

Note: ESI: Positive and negative ion detection mode; RT: Retention time; QC: Quality control;QCRSD:Relative standard deviation of QC samples.

Table S3. OPLS-DA model evaluation parameters for each comparison group of targeted energy metabolomics

Sample Comparison Groups	R2X(cum)	R2Y(cum)	Q2(cum)	RMSEE	pre	ort	pR2Y	pQ2	R2 intercept	Q2 intercept
NC_HC_HCP	0.747	0.995	0.944	0.0911	1	2	0.01	0.005	0.903	-0.037

Note: NC: Normoxia control group; HC: Hormoxia control group; HCP: Hypoxia combined with PT2385 group; R2X and R2Y represent the interpretation rate of the model for X and Y matrices; Q2: indicates the predictive ability of the model; The closer R2 and Q2 are to 1, the more stable and reliable the model is. Q2 > 0.5 indicates the better predictive ability of the model, and Q2 < 0.5 indicates the worse predictive ability of the model. RMSEE: root mean square error of the estimate; pre represents predictive group fraction for modeling, ort represents orthogonal group fraction for modeling. pR2Y: p value of R2Y, pQ2: p value of Q2; R2 intercept and Q2 intercept: Indicates the intercept between R2 and Q2 regression lines and the Y-axis.

Table S4 Baseline features of chronic hypoxic lung disease patients with or without PH

Parameter		Chronic hypoxic lung disease with pulmonary hypertension (n = 104)	Chronic hypoxic lung disease without pulmonary hypertension (n = 65)	*P*-value
**Demographics and clinical features**				
Age (years)		70.00(64.25,78.00)	57.00(49.00,68.50)	0.000
Sex	Male	63.5%	64.6%	0.880
	Female	36.5%	35.4%	
Race	Han	67.3%	69.2%	0.771
	Tibet	12.5%	13.9%	
	Hui	15.4%	13.8%	
	Others	4.8%	3.1%	
Altitude(m)		2295.00(2295.00,2800.00)	2295.00(2295.00,3134.50)	0.088
**Laboratory features**				
Hemoglobin (g/dL)		171.64±34.25	171.00±30.82	0.902
Hematocrit		52.35（44.30,61.43）	48.50(45.80,57.05)	0.474

Creatine kinase	55.00(41.00,75.00)	75.50(45.50,116.25)	0.028
Creatine kinase-MB	12.00(9.00,16.00)	12.00(9.25,13.00)	0.260
Lactate dehydrogenase（U/L）	220.00(187.00,277.25)	205.00(177.50,235.00)	0.018
Lactate dehydrogenase-1	44.00(35.25,64.50)	38.00(31.00,47.25)	0.008
Troponin I	7.87(3.67,24.58)	1.23(0.03,2.59)	0.000
Brain natriuretic peptide	179.00(65.25,572.50)	38.00(19.00,65.00)	0.000
Serum iron	9.75(6.85,15.38)	15.2(10.45,20.35)	0.000
Transferrin saturation (TSAT)	0.19(0.13,0.35)	0.28(0.20,0.39)	0.004
Ferritin (ng/mL)	200.20(106.00,360.45)	247.80(107.25,557.15)	0.124
High sensitive C-reactive protein	0.64(0.26,2.57)	0.26(0.09,1.12)	0.001
**Echocardiography**			
Pulmonary arterial systolic pressure(PASP)	60.00(47.00,76.00)	30.00(30.00,30.00)	0.000
Right atrial transverse diameter(mm)	41.00(34.00,47.000	34.00(31.00,37.00)	0.000
Transverse diameter of right	32.00(28.25,37.00)	28.00(26.00,29.00)	0.000

ventricle(RVTD,mm)			
Right ventricular wall thickness(mm)	5.00(4.00,5.00)	4.00(4.00,5.00)	0.000
Outflow tract of right ventricle(mm)	30.50(28.00,36.00)	29.00(27.25,31.00)	0.007
Left ventricular ejection fraction (LVEF%)	66.00(62.00,70.00)	65.00(62.00,69.00)	0.621

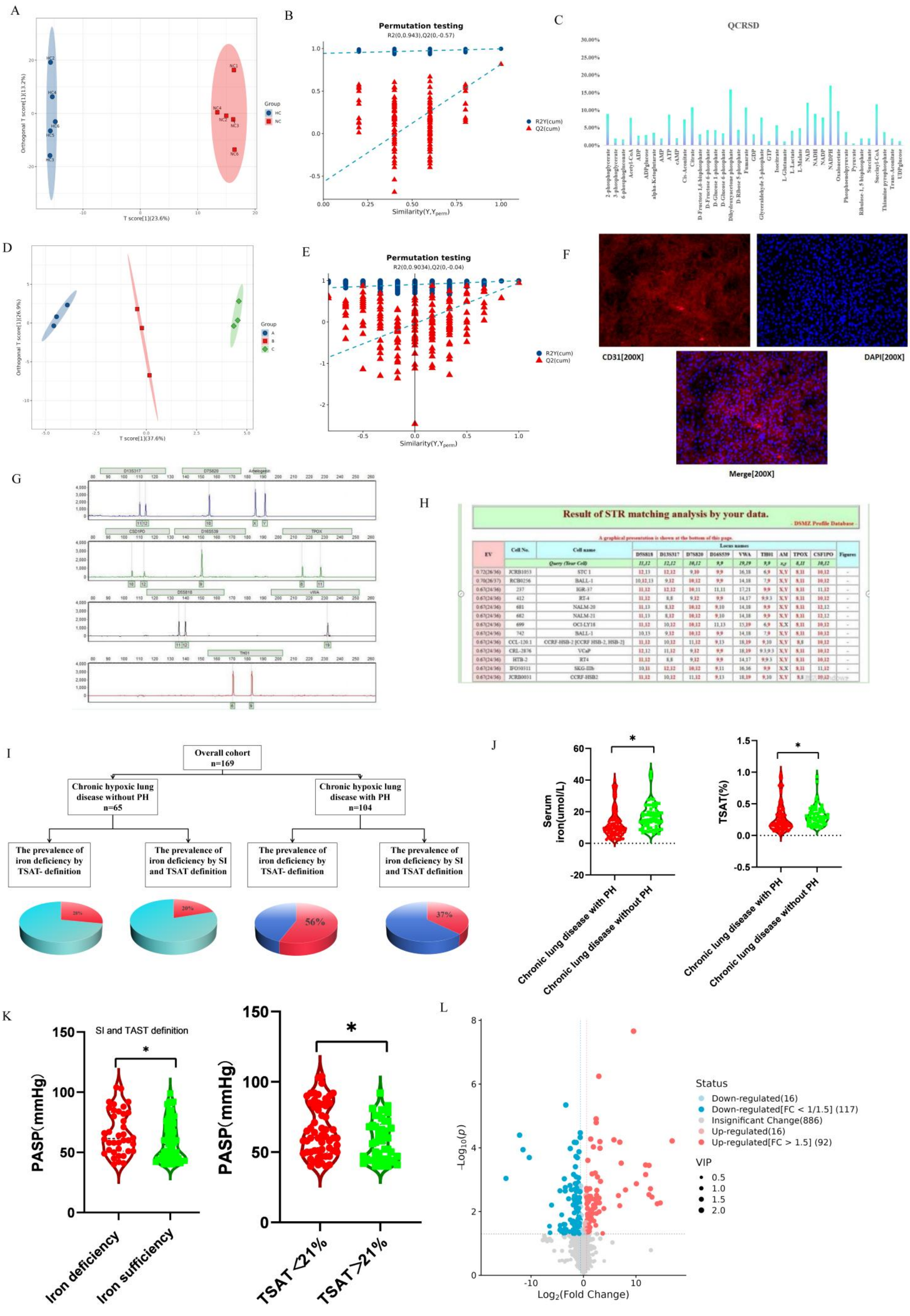

A
T score[1](23.6%)
Orthogonal T score[1](13.2%)
Group
HC
NC
B
Permutation testing
R2(0,0.943),Q2(0,-0.57)
R2Y(cum)
Q2(cum)
Similarity(Y,Yperm)
C
QCRSD
D
T score[1](37.6%)
Orthogonal T score[1](26.9%)
Group
A
B
C
E
Permutation testing
R2(0,0.9034),Q2(0,-0.04)
R2Y(cum)
Q2(cum)
Similarity(Y,Yperm)
F
CD31[200X]
DAPI[200X]
Merge[200X]
G
D13S317
D7S820
Amelogenin
CSF1PO
D16S539
TPOX
D5S818
vWA
TH01
H
Result of STR matching analysis by your data.
- DSMZ Profile Database -
I
Overall cohort
n=169
Chronic hypoxic lung disease without PH
n=65
Chronic hypoxic lung disease with PH
n=104
The prevalence of iron deficiency by TSAT- definition
The prevalence of iron deficiency by SI and TSAT definition
The prevalence of iron deficiency by TSAT- definition
The prevalence of iron deficiency by SI and TSAT definition
28%
20%
56%
37%
J
Serum iron(umol/L)
TSAT(%)
Chronic lung disease with PH
Chronic lung disease without PH
Chronic lung disease with PH
Chronic lung disease without PH
K
SI and TAST definition
PASP(mmHg)
Iron deficiency
Iron sufficiency
PASP(mmHg)
TSAT<21%
TSAT>21%
L
Status
Down-regulated(16)
Down-regulated[FC < 1/1.5] (117)
Insignificant Change(886)
Up-regulated(16)
Up-regulated[FC > 1.5] (92)
VIP
0.5
1.0
1.5
2.0
Log2(Fold Change)
-Log10(p)

Figure S1: The metabolomics datasets, cellular validation results, and retrospective clinical patient data analyses. (A, B) The OPLS-DA model score plot and permutation test plot of two comparison groups (HC.vs.NC); (C) Relative standard deviation (RSD) results of the to-be-measured substances in QC samples(The data of energy metabolites with RSD less than 30% in the sample are stable and reliable); (D, E) The OPLS-DA model score plot and permutation test plot for three comparison groups of targeted energy metabolomics; (F) CD31 fluorescence identification of RPAEC (red fluorescence in the picture is CD31 positive, the positivity rate is >90%, i.e.: the purity of the cells is >90%); (G) Genotyping results of Amelogenin locus in HPAECs; (H) EXPASY database comparison results; (I) Prevalence of iron deficiency in patients with PH due to chronic lung disease. (J, K) Differences in iron metabolism between two groups of patients with chronic lung disease with or without PH; (L) Comparison group HC.vs. NC volcano plot (*p*-value + VIP screening for differential metabolites, red indicates up-regulation, blue indicates down-regulation, number of categories in the figure notes). *$p < 0.05$.

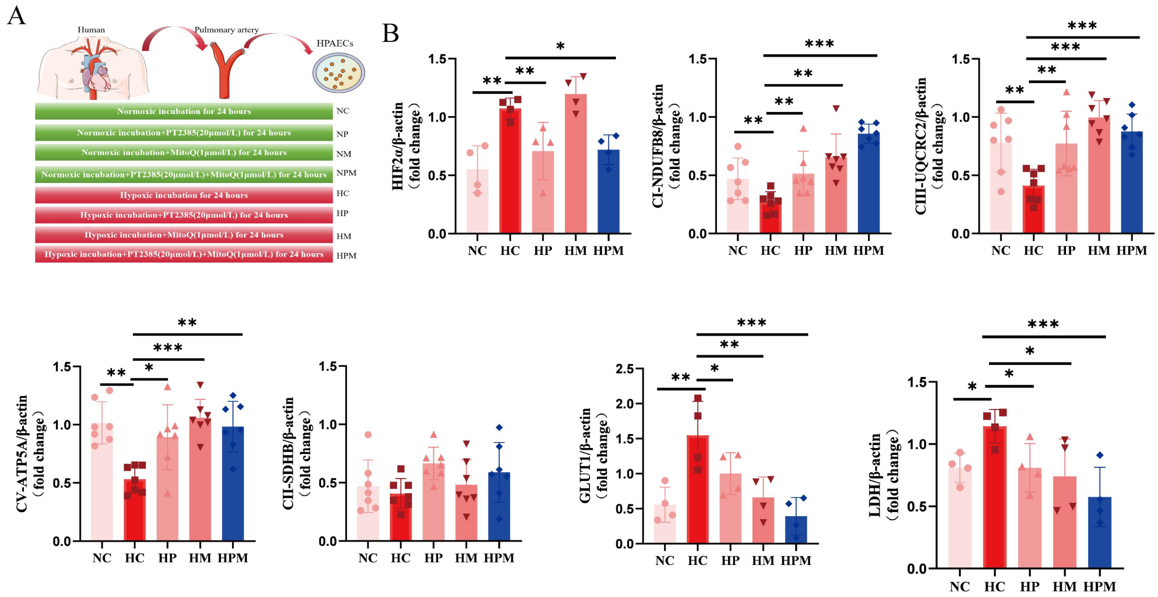

Figure S2: The detailed grouping diagram of HPAECs experiments and quantitative data of Western blot analysis for pharmacological intervention in HPAECs. (A) Detailed grouping diagram of HPAECs experiments. (B) The quantitative data of Western blot analysis for pharmacological intervention in HPAECs. NC: Normoxia control group; HC: Hypoxia control group; HP: Hypoxia combined with PT2385 group; HM: Hypoxia combined with MitoQ group; HPM: Hypoxia combined with PT2385 and MitoQ group. The data are presented as mean ± SD; * $p < 0.05$; ** $p < 0.01$; *** $p < 0.001$.

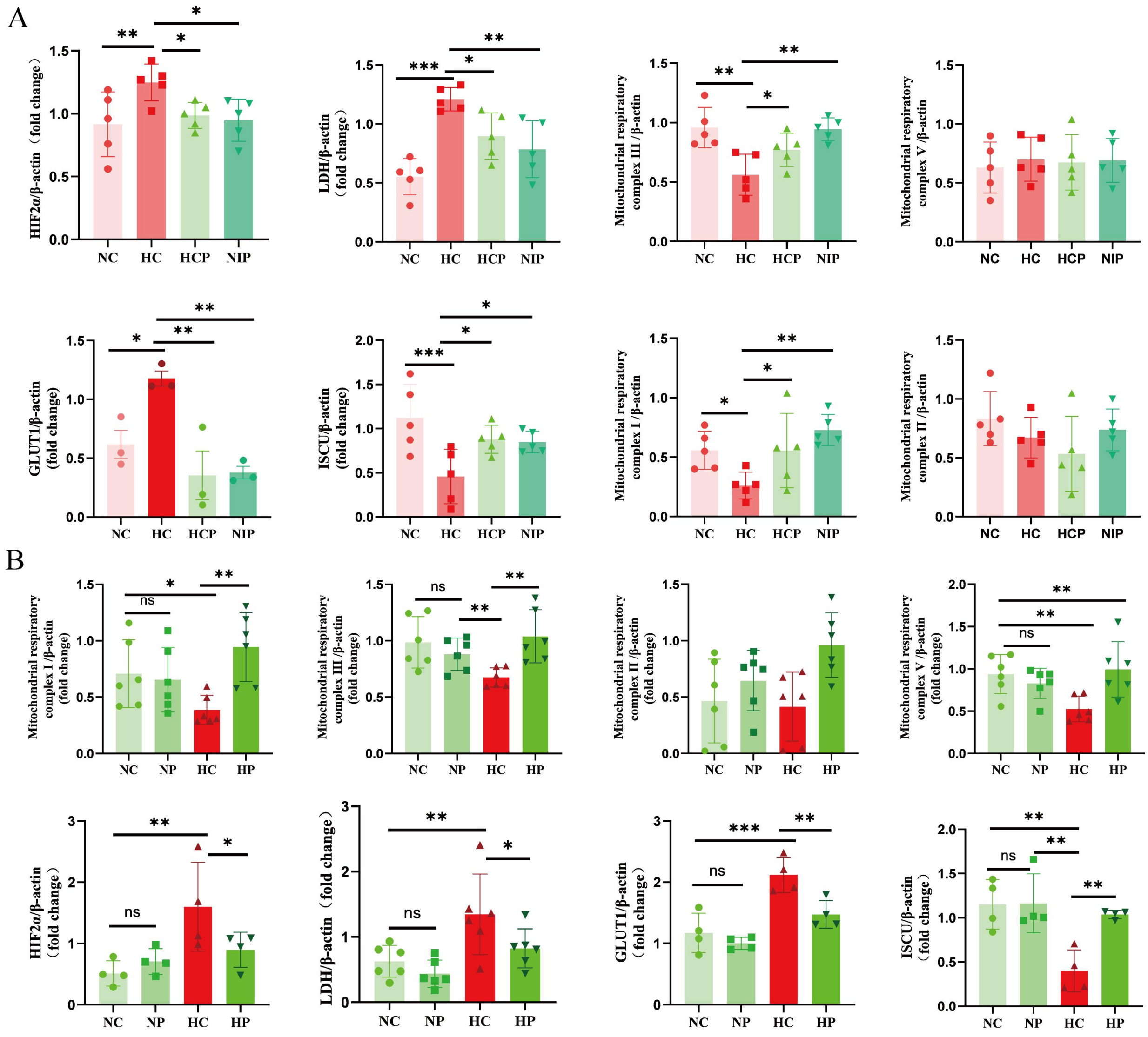

Figure S3: The quantitative results of Western blot analysis for PT2385 intervention in mouse lung tissues (A) and RPAECs (B), Corresponding to Figure 9C and D in the main text; NC (Normoxic control group), HC (Hypoxia control group), HCP (Hypoxia combined with PT2385 group), NIP (PT2385 intervention for 1 week after 4-week hypoxia exposure), NP (Normoxia combined with PT2385 group), and HP (Hypoxia combined with PT2385 group); The data are presented as mean ± SD; * $p < 0.05$; ** $p < 0.01$; *** $p < 0.0001$; ns: No statistical significance.
