# Supplementary material for: Iron Replacement Attenuates Hypoxic Pulmonary Hypertension by Remodeling Energy Metabolism via Regulating the HIF2α/Mitochondrial Complex I, III/ROS Axis
Source: Biomolecules. 2025 May 21;15(5):742. doi: 10.3390/biom15050742 (PMC12109292; doi:10.3390/biom15050742)

This file is the Original Images for Western Blot.

The marker applied for Western Blot in this study was obtained from Thermo Fisher Scientific (no. 26617), and the specific molecular weights are shown in the figure below.

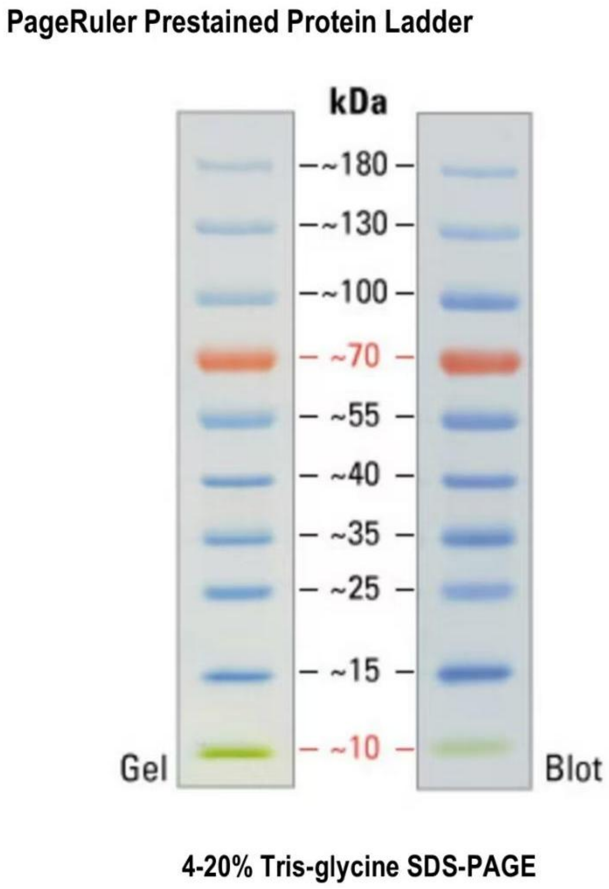

Figure 4G(The image on the left is HIF2α, and the corresponding housekeeping Protein is on the right):

HIF2α： β-actin：

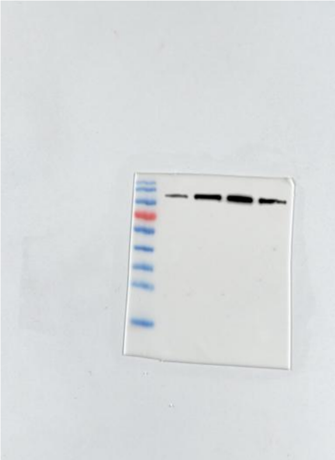
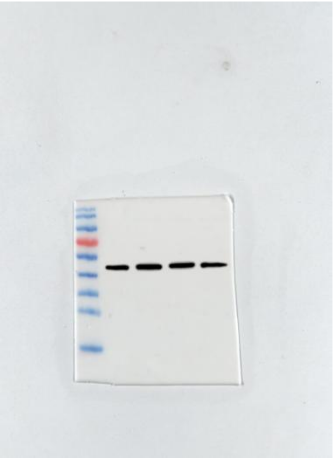

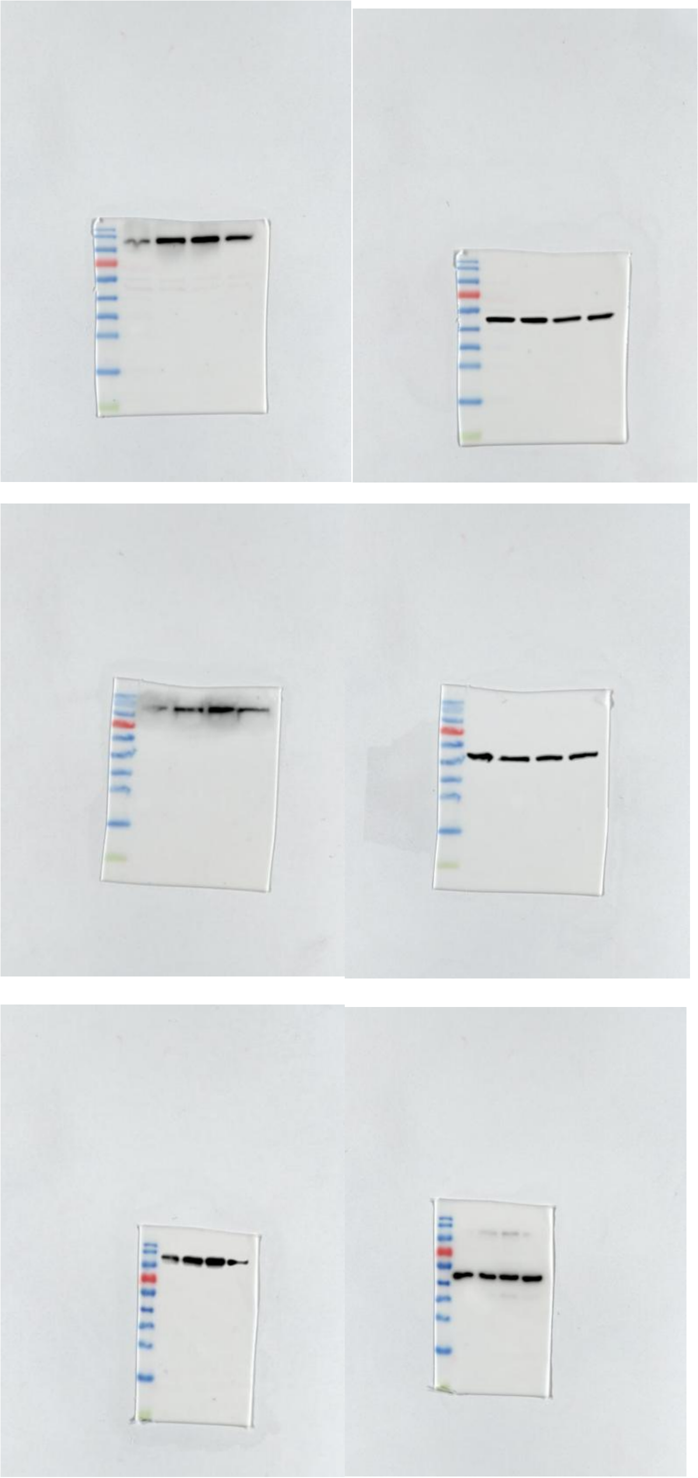

Figure 5F(The image on the left is HIF2α, and the corresponding housekeeping Protein is on the right)：

HIF2α：　　　　　　　　　　　β-actin：

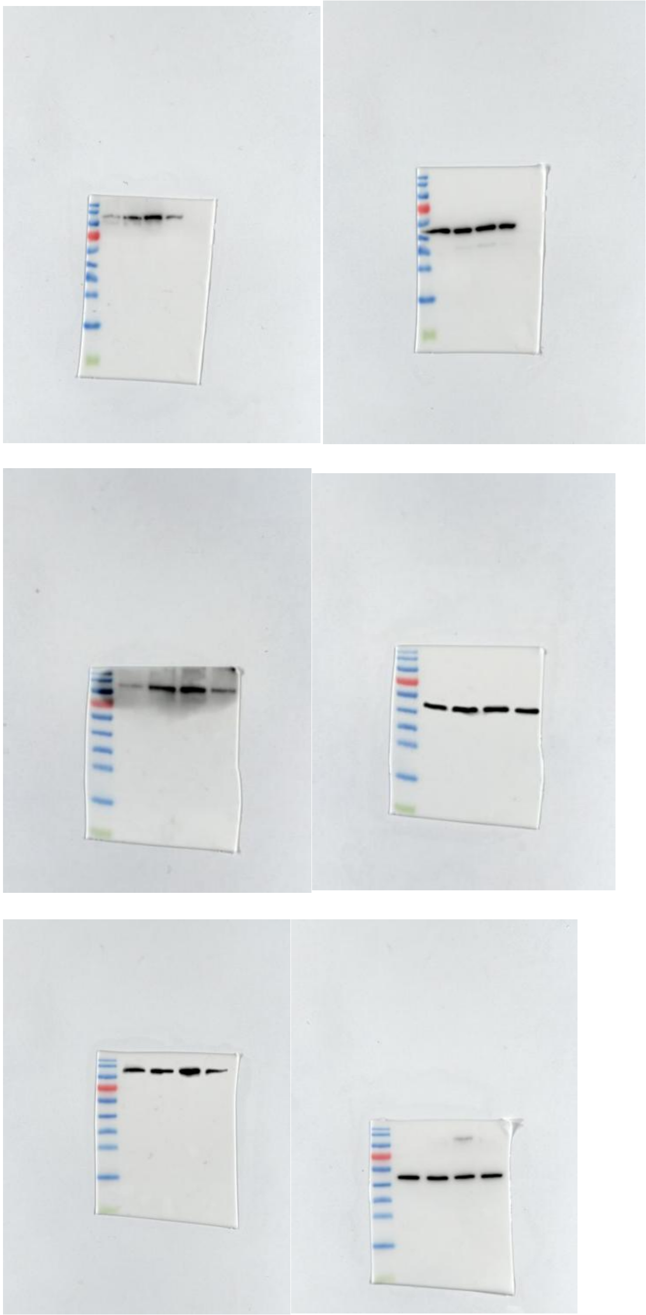

Figure 8F(The image on the left is mitochondrial complex, and the corresponding housekeeping Protein is on the right):

Mitochondrial complex: β-actin：

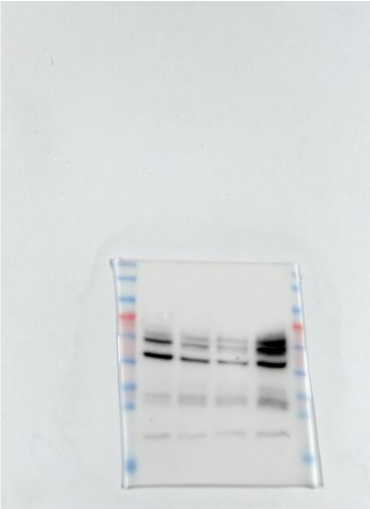
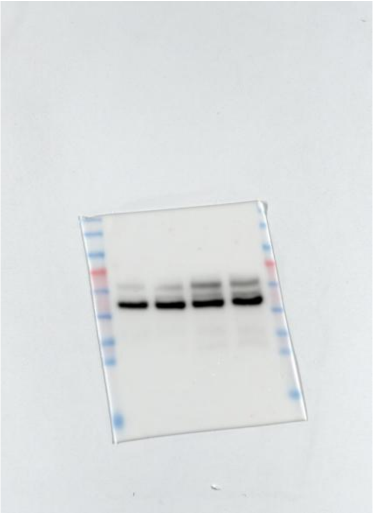
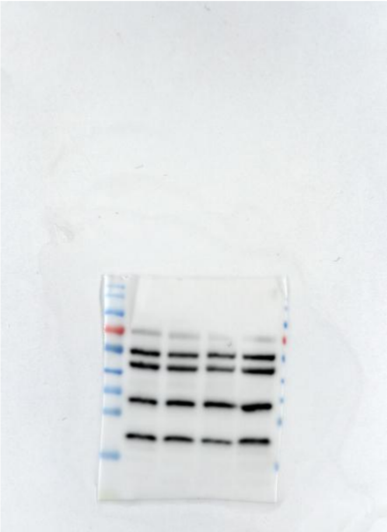
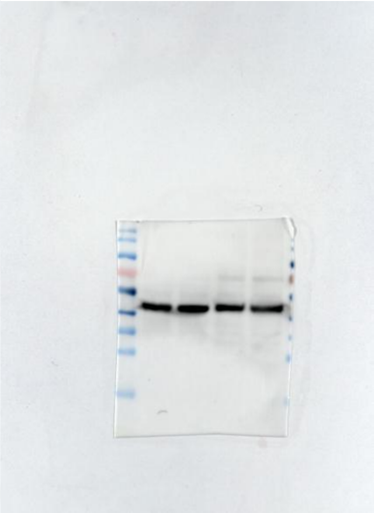
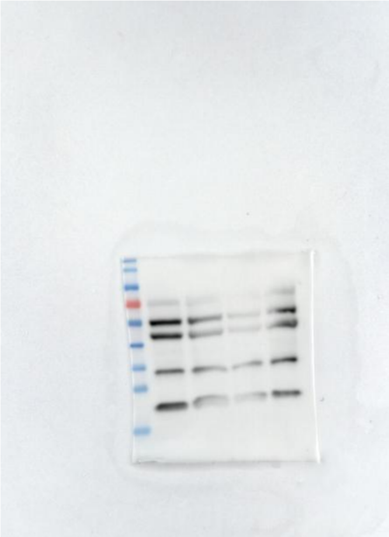
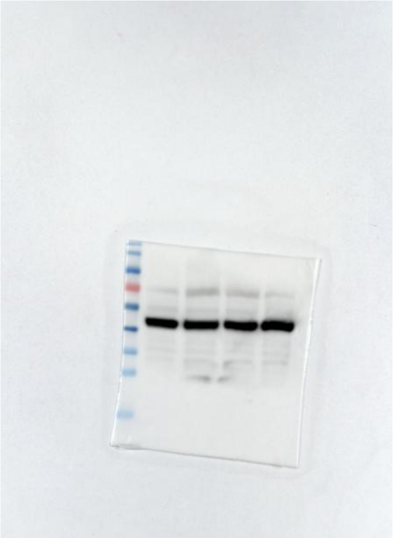

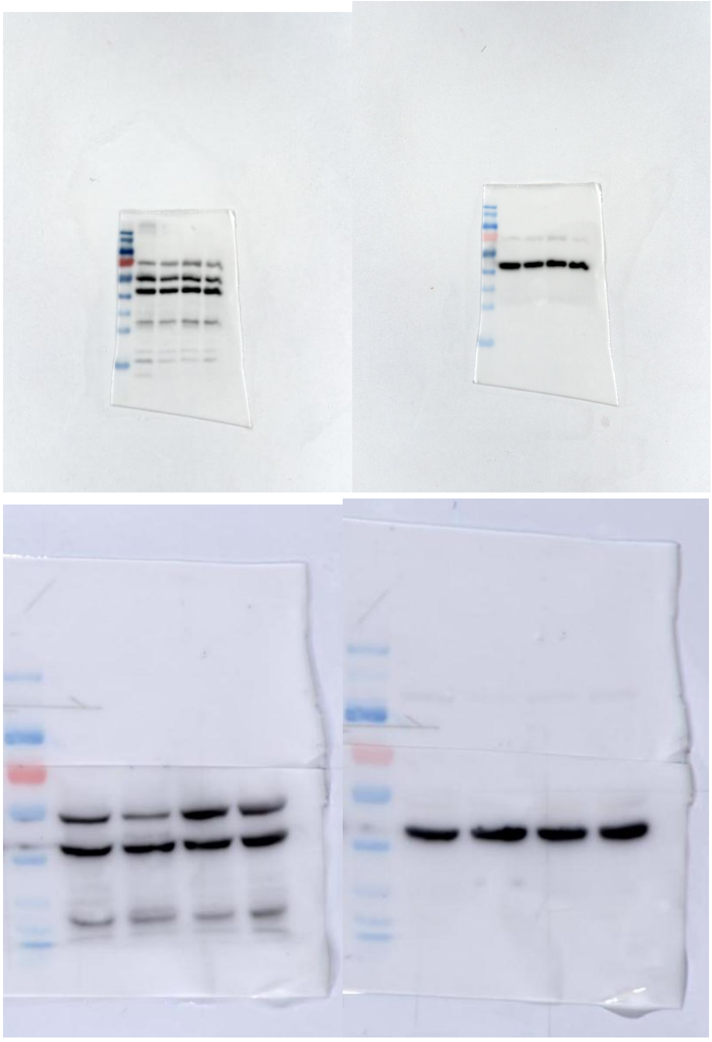

Figure 9C(The image on the left is HIF2α, and the corresponding housekeeping Protein is on the right):

HIF2α:　　　　　　　β-actin:

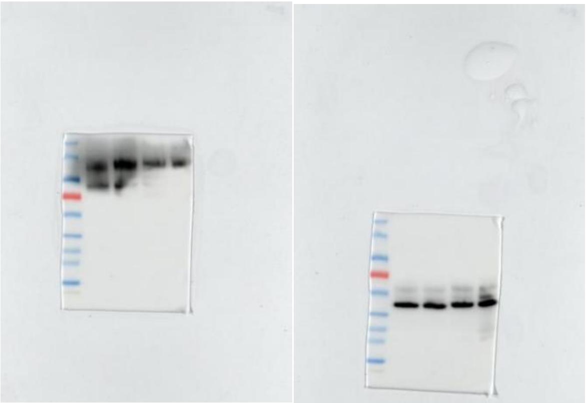

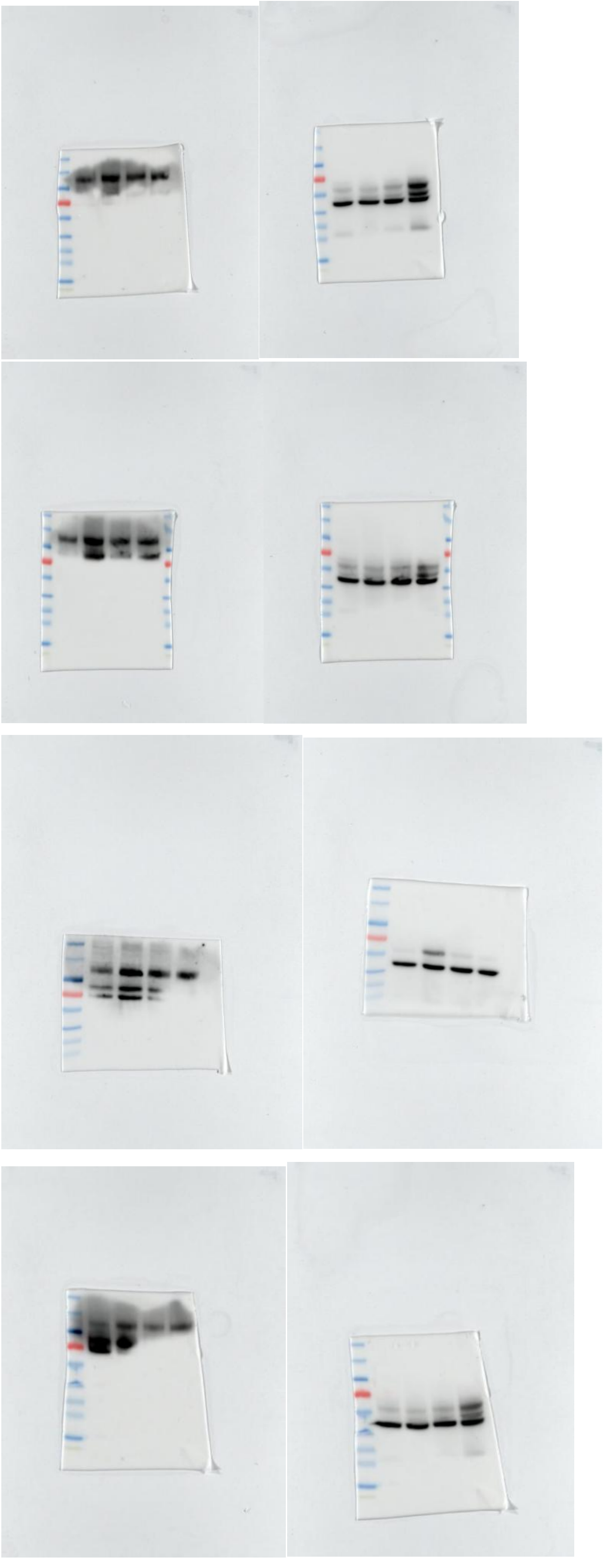

Figure 9C(The image on the left is mitochondrial complexes, and the corresponding

housekeeping Protein is on the right):

Mitochondrial complexes： β-actin：

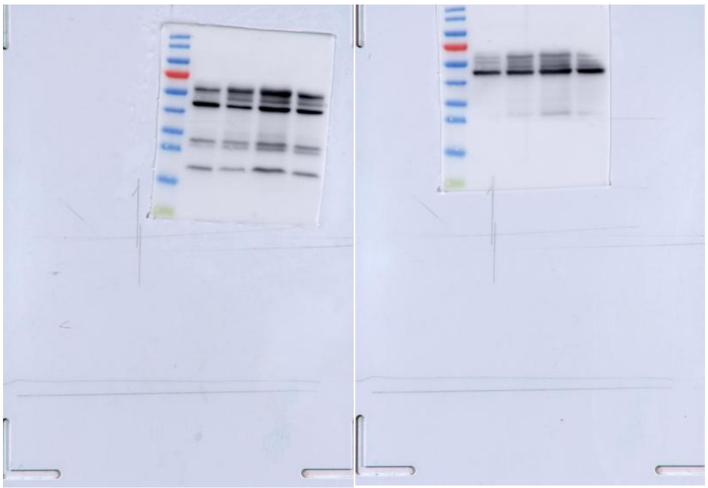

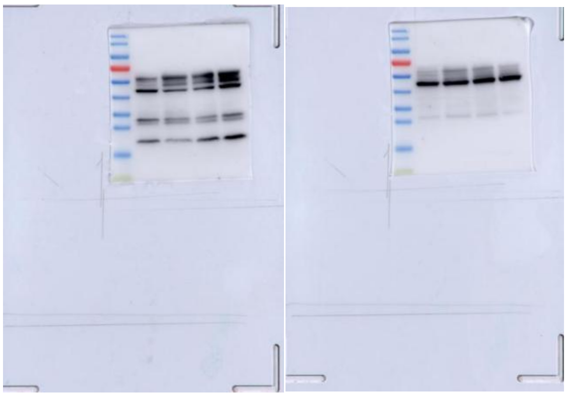

Mitochondrial complexes: GAPDH:

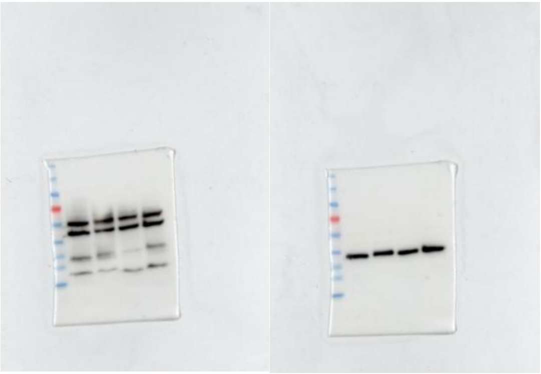

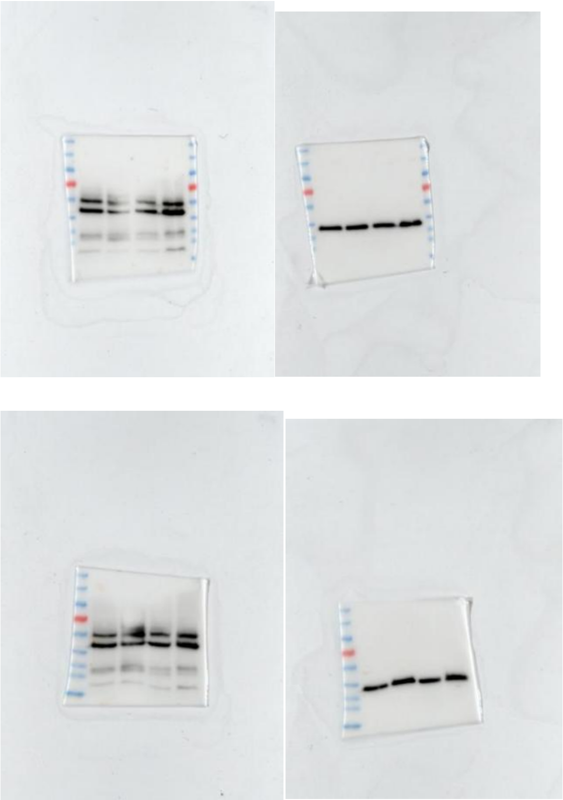

Figure 9C(The image on the left is GLUT1, and the corresponding housekeeping Protein is on the right):

GLUT1: β-actin：

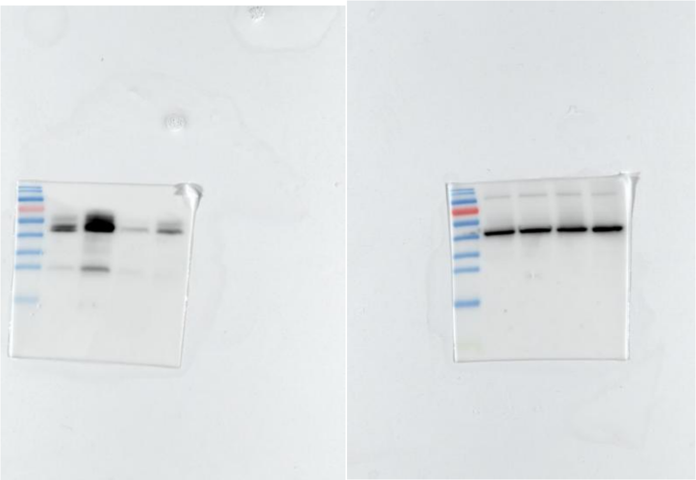

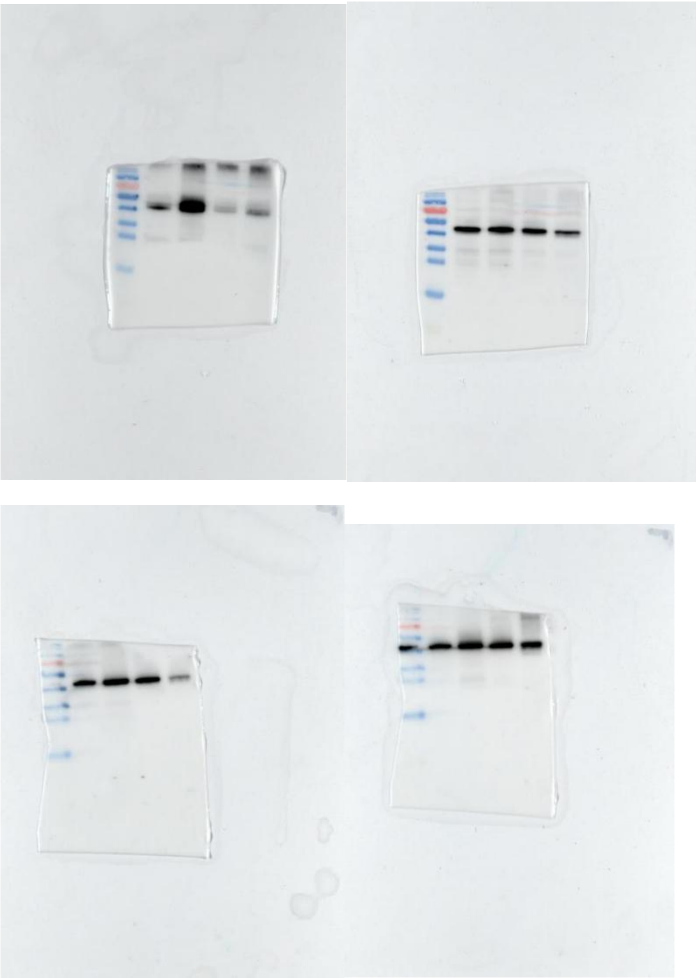

Figure 9C(The image on the left is LDH, and the corresponding housekeeping Protein is on the right):

LDH：   β-actin：

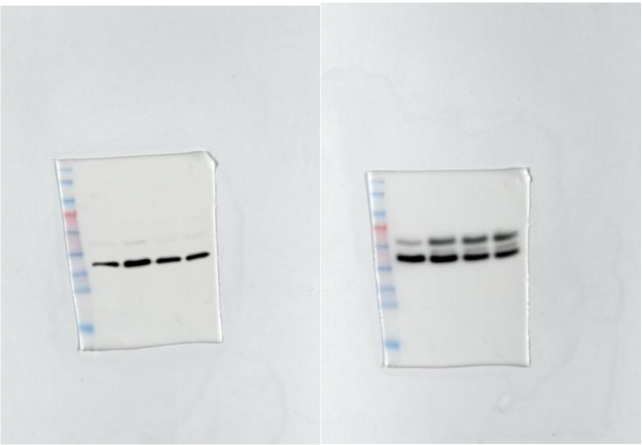

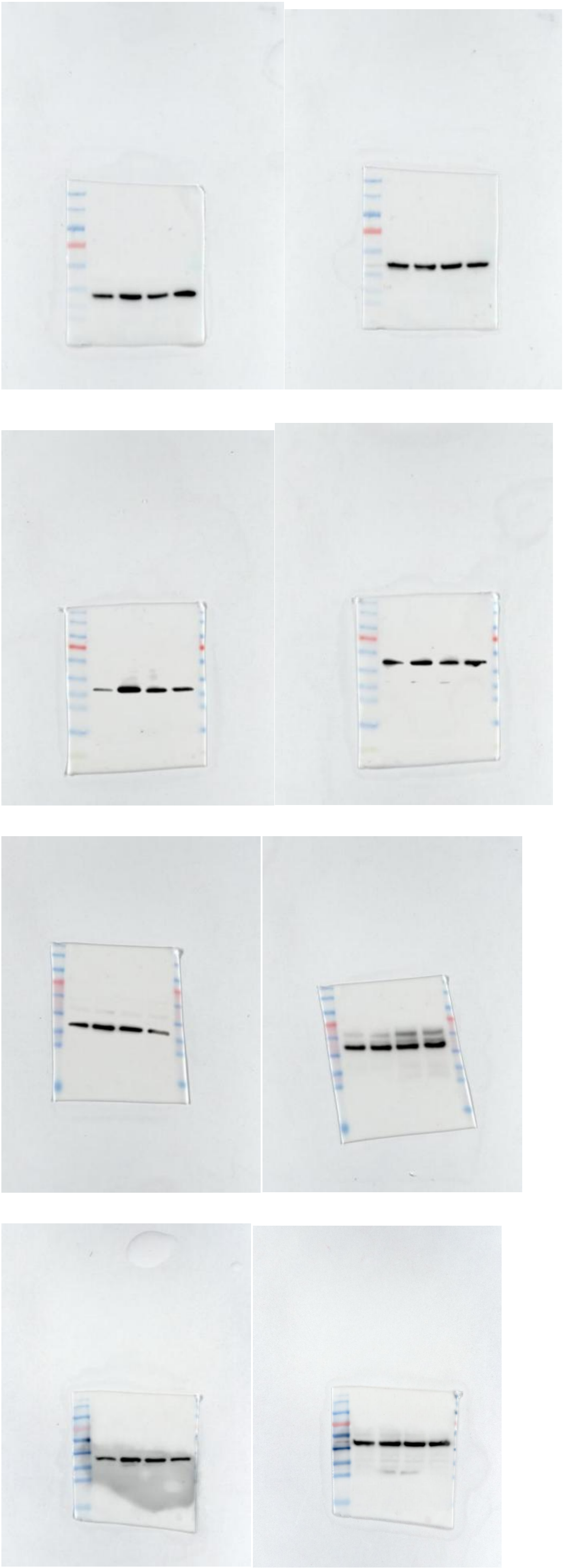

Figure 9C(The image on the left is ISCU, and the corresponding housekeeping Protein is on the right):

ISCU: β-actin:

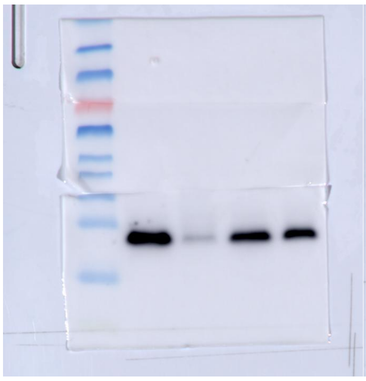
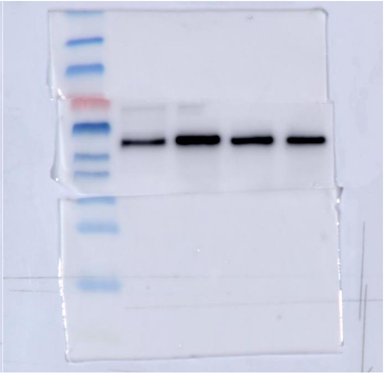
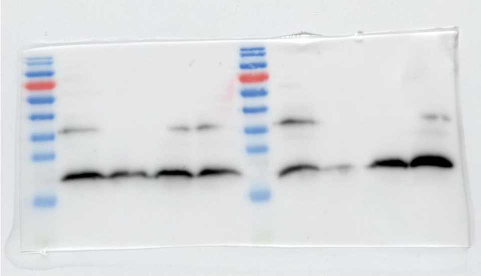
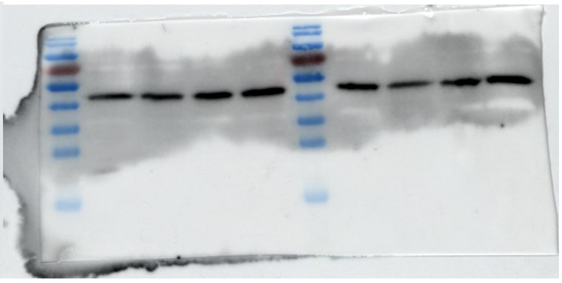

ISCU: GAPDH:

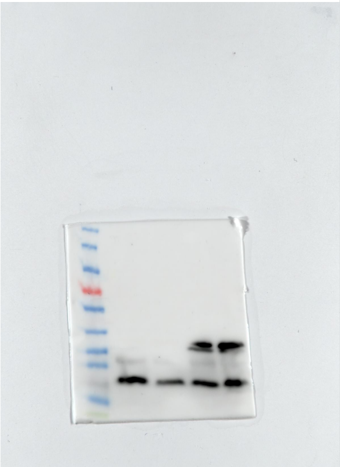
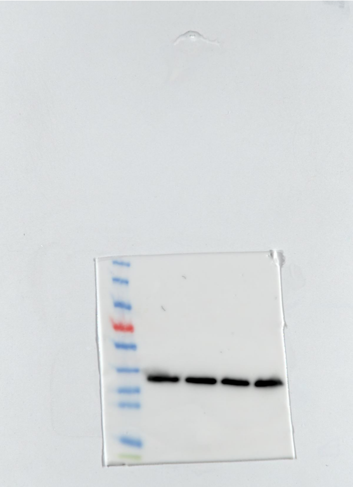
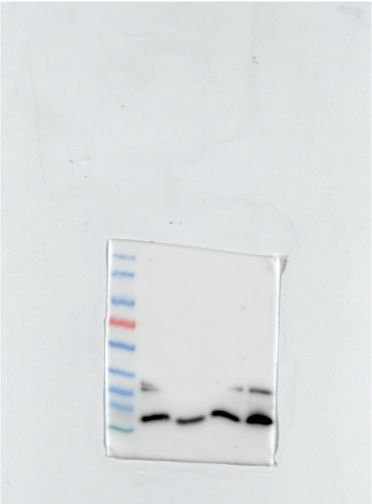
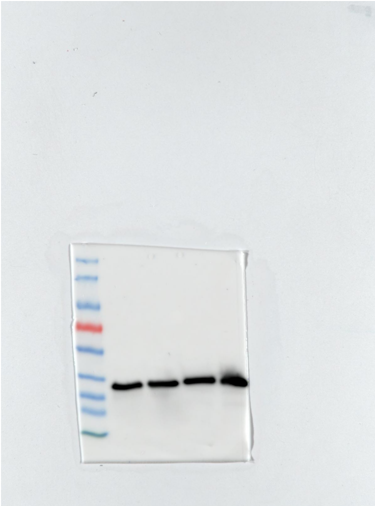

Figure 9D(The image on the left is HIF2α, and the corresponding housekeeping Protein is on the right):

HIF2α：　　　　　β-actin：

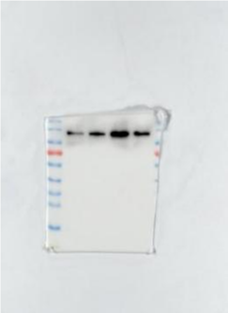
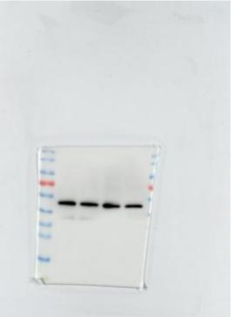

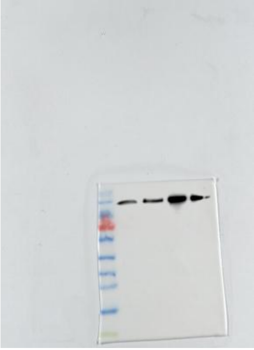
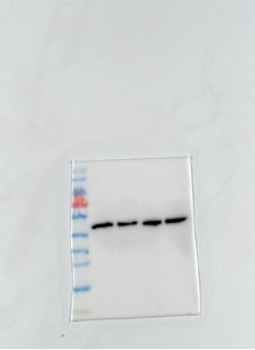

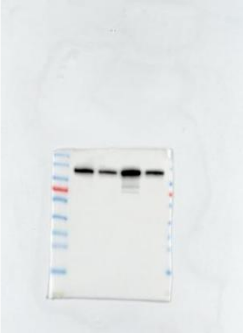
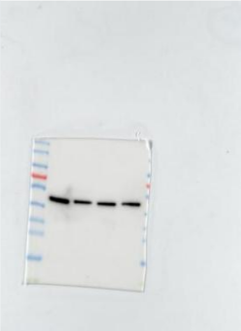

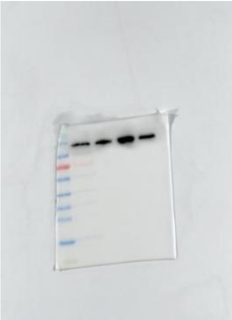
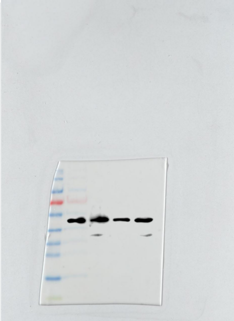

Figure 9D(The image on the left is mitochondrial complex, and the corresponding housekeeping Protein is on the right):

Mitochondrial complex:　　　　β-actin：

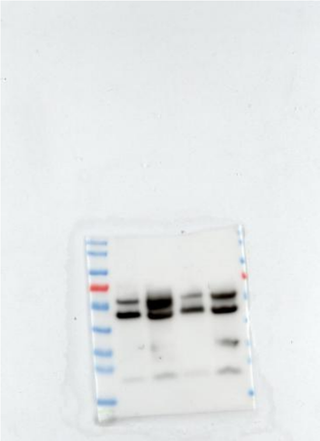
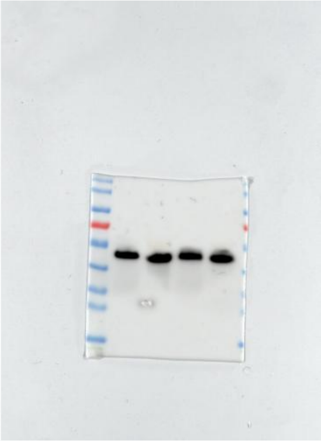
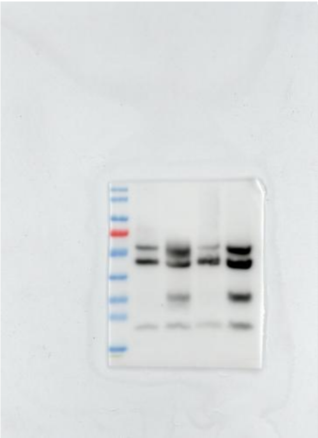
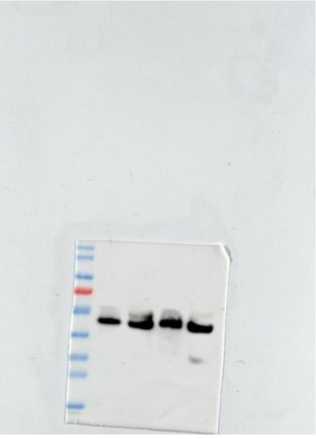
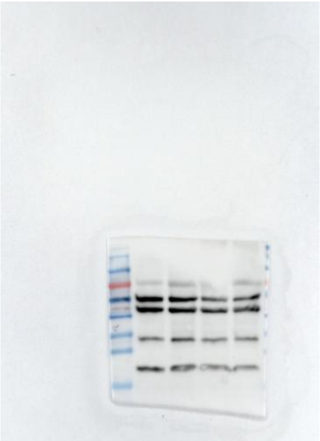
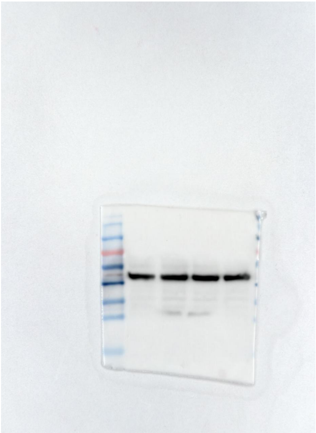
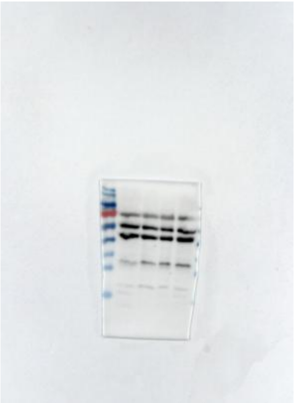
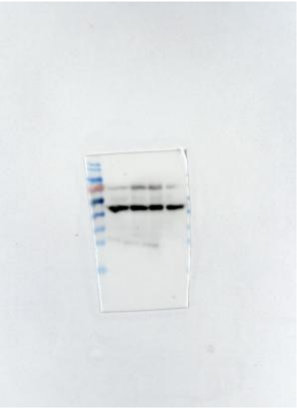

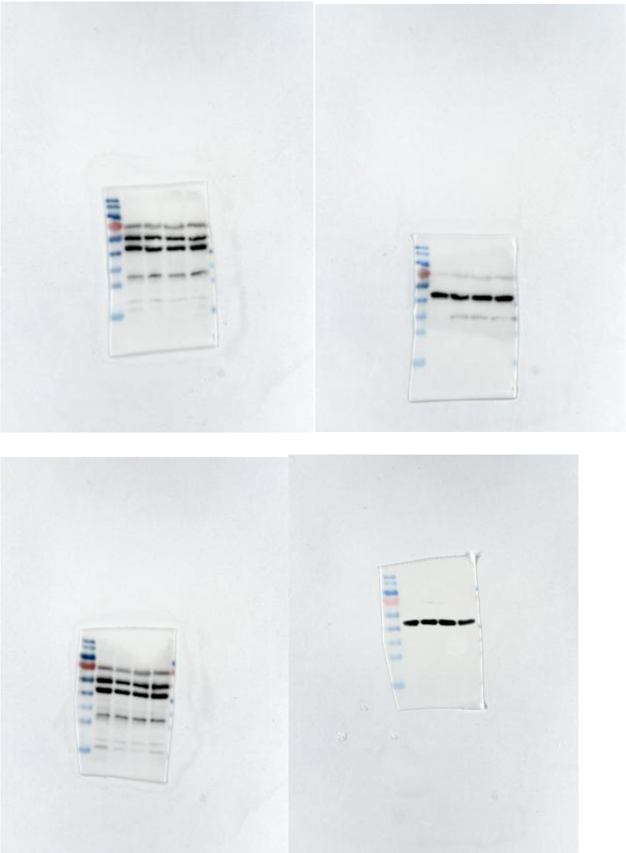

Figure 9D(The image on the left is GLUT1, and the corresponding housekeeping Protein is on the right):

GLUT1: β -actin：

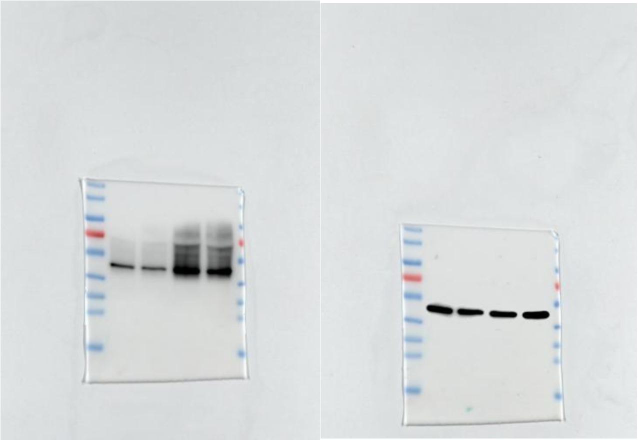

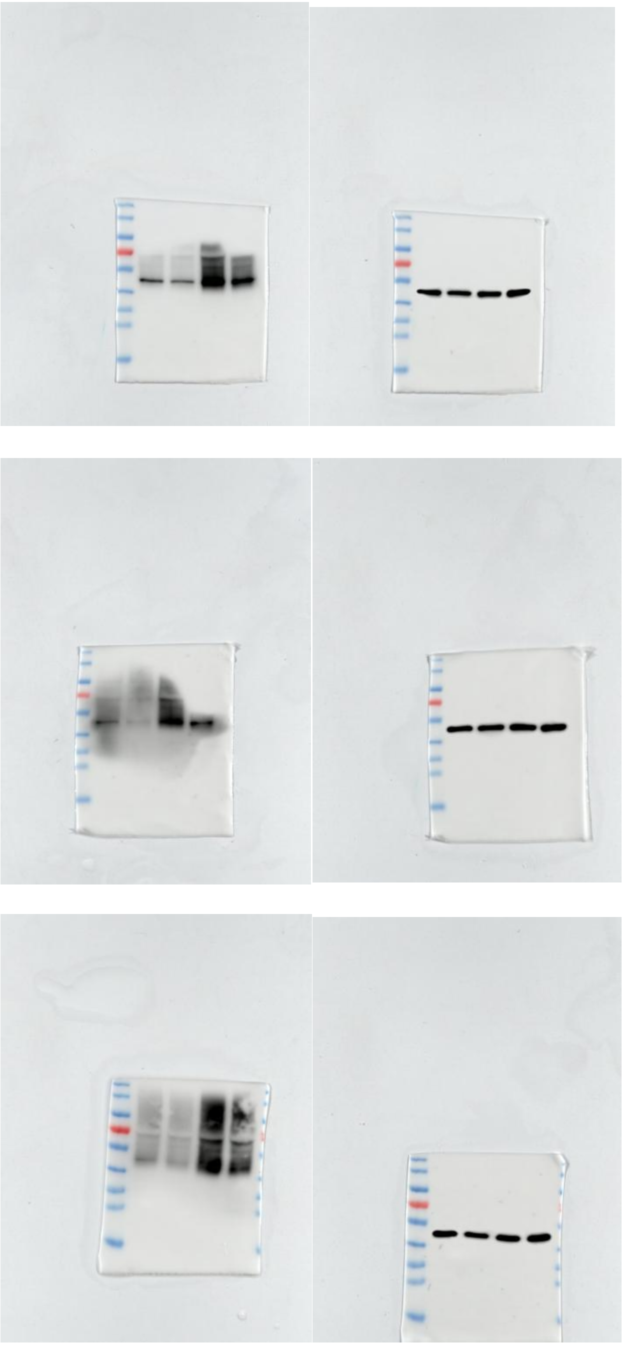

Figure 9D(The image on the left is LDH, and the corresponding housekeeping Protein is on the right):

LDH: β-actin：

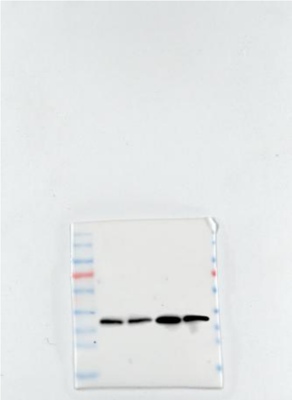
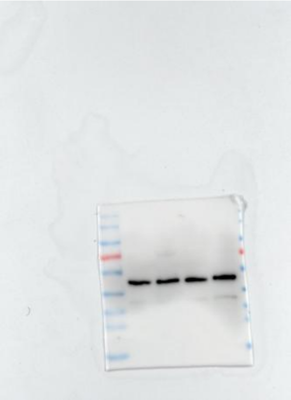
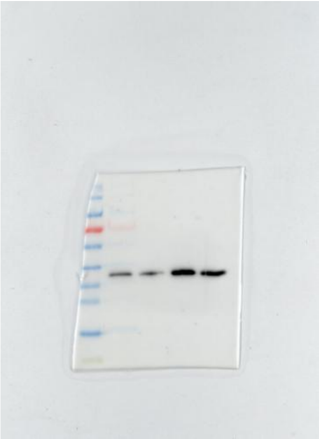
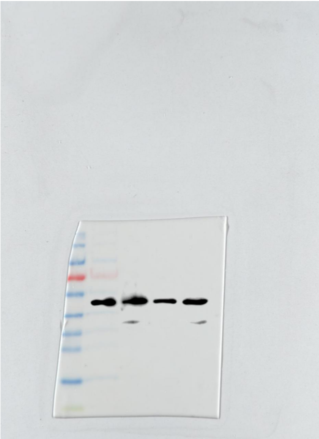
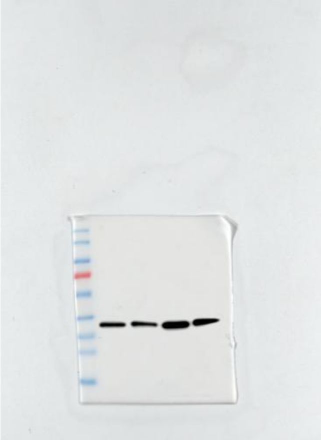
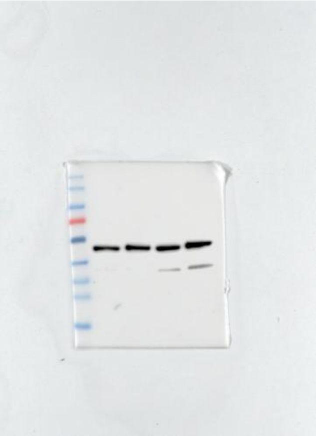
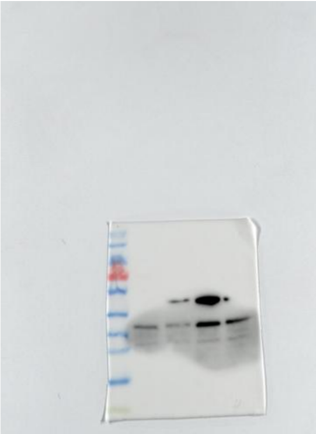
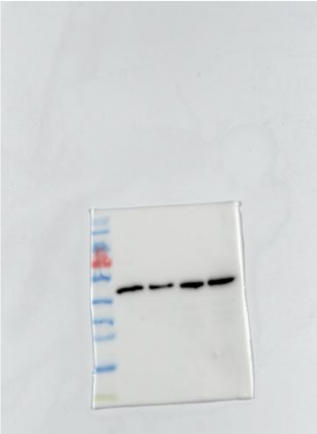

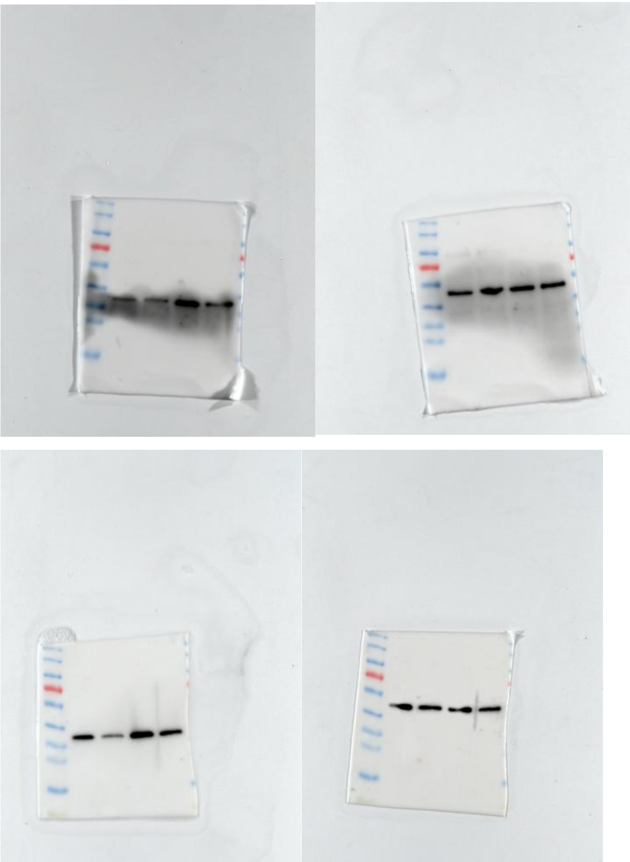

Figure 9D(The image on the left is ISCU and the corresponding housekeeping Protein is on the right):

ISCU:

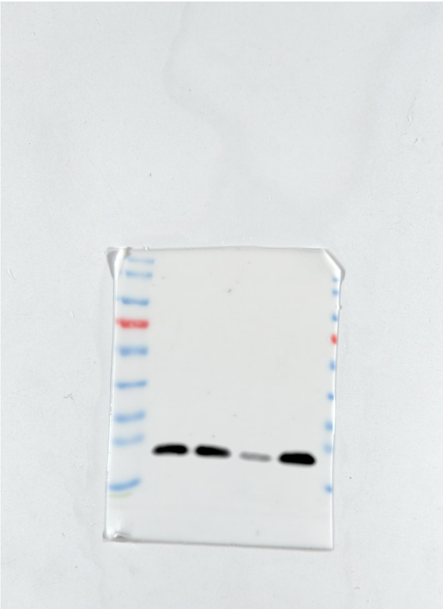

β-actin:

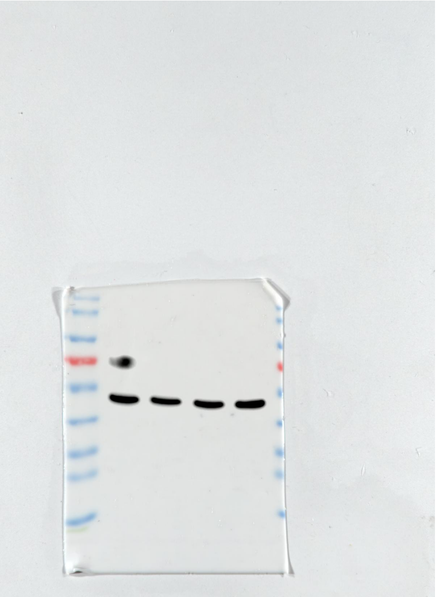

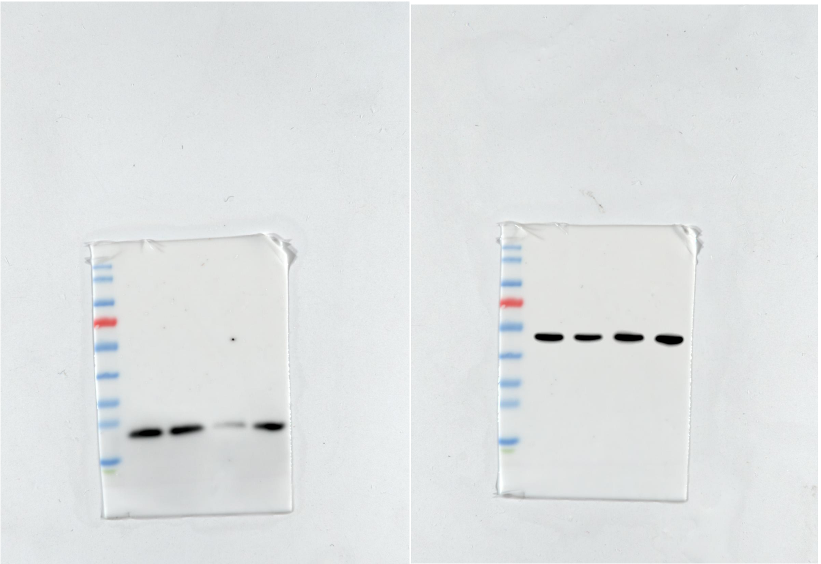

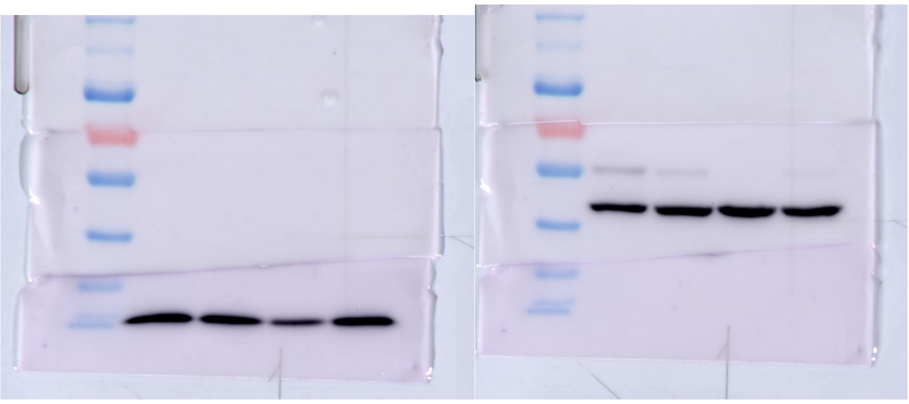

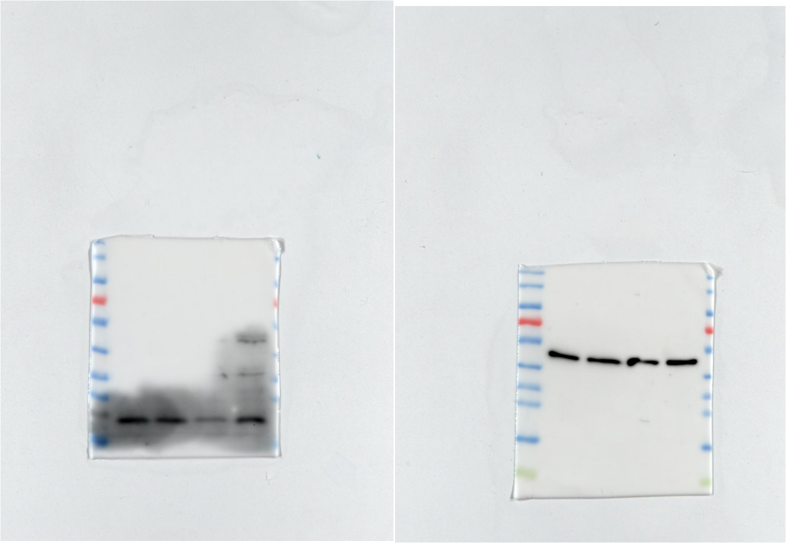

Figure 10L(The image on the left is HIF2α, and the corresponding housekeeping Protein is on the right):

HIF2α: **β-actin：**

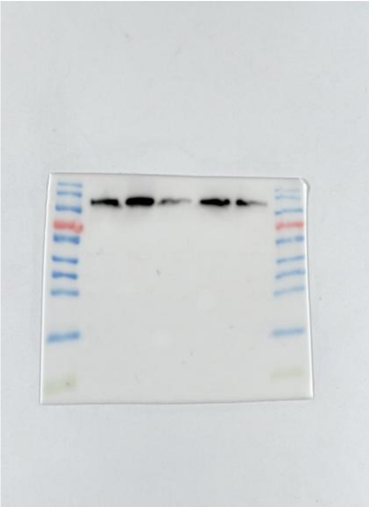
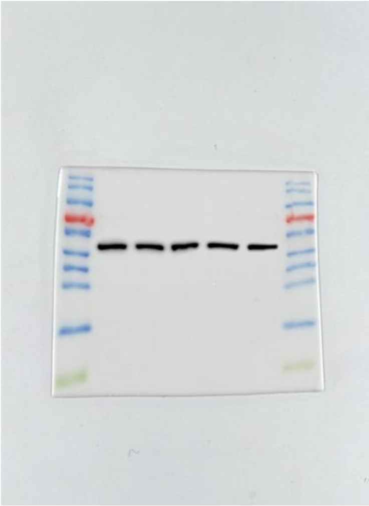
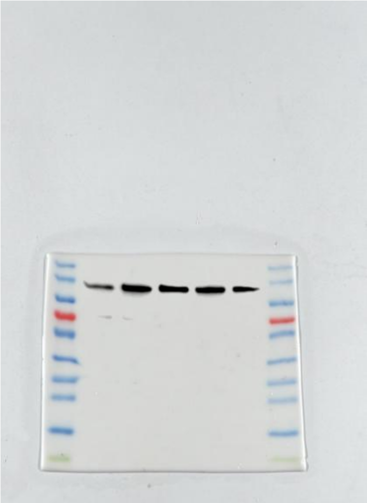
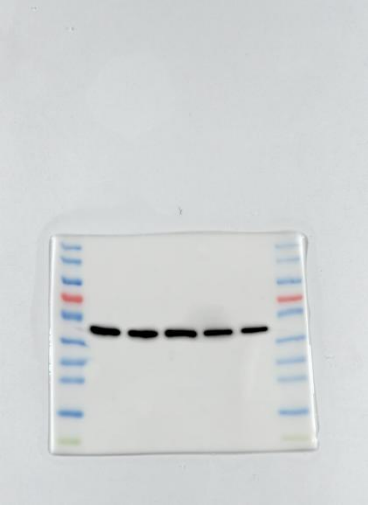
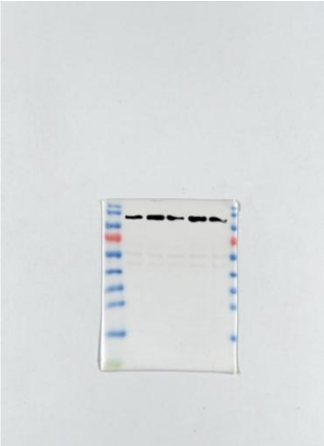
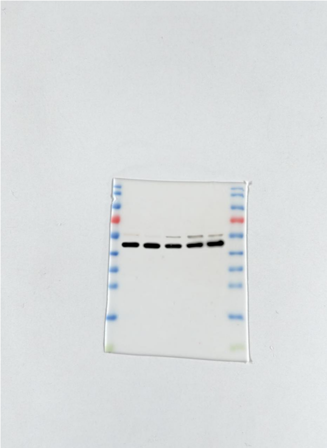

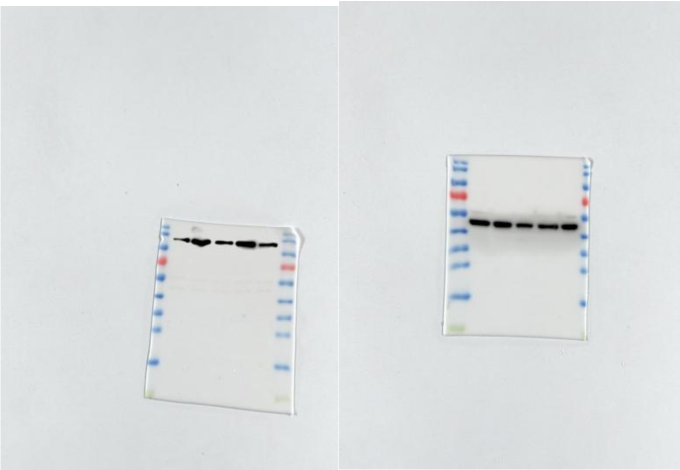

Figure 10L(The image on the left is mitochondrial complexes, and the corresponding housekeeping Protein is on the right):

Mitochondrial complexes： **β-actin：**

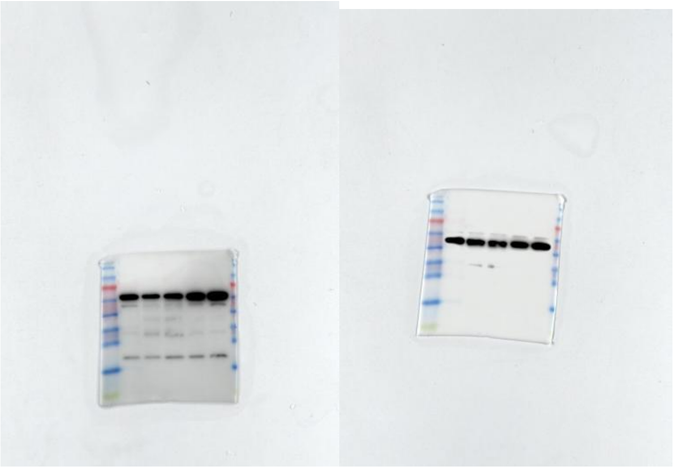

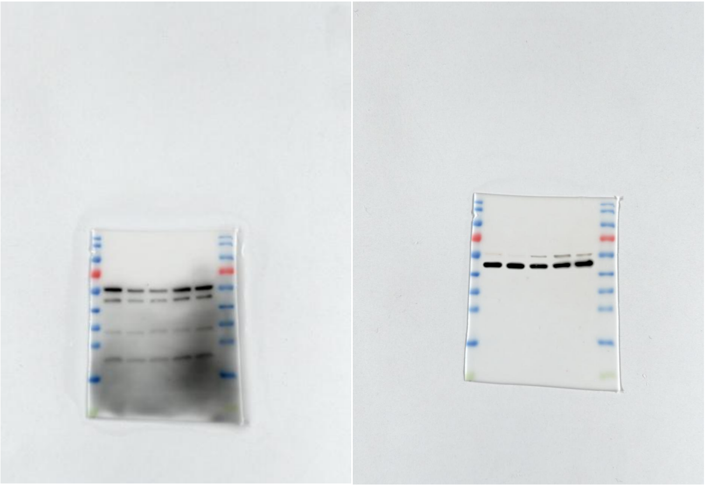

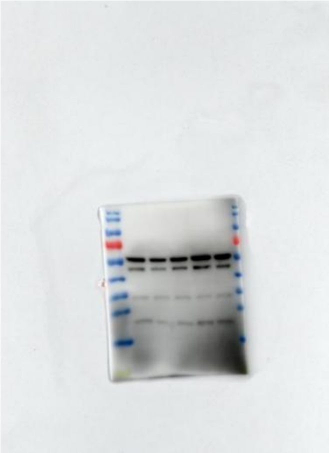
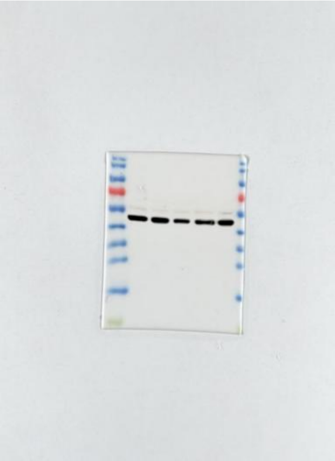
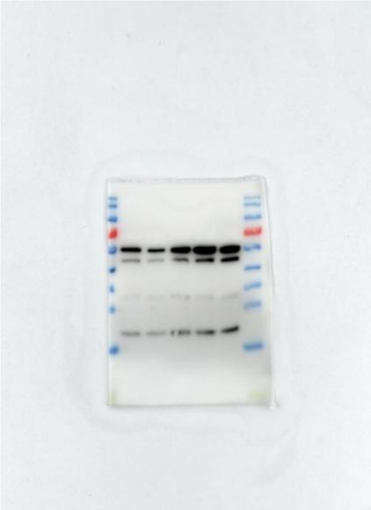
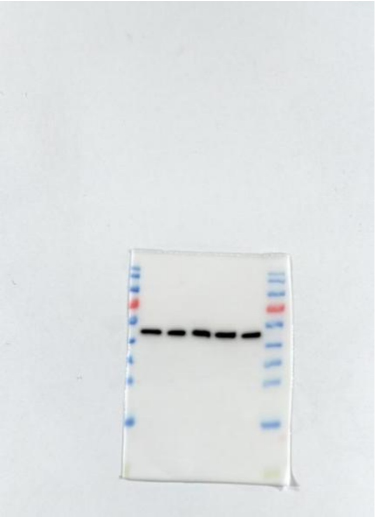
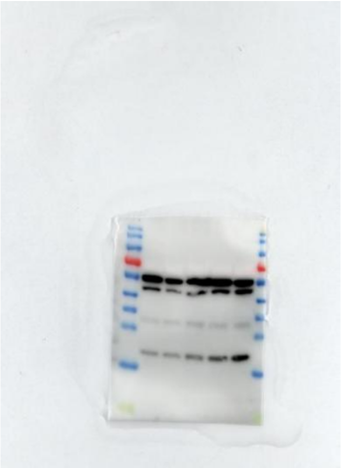
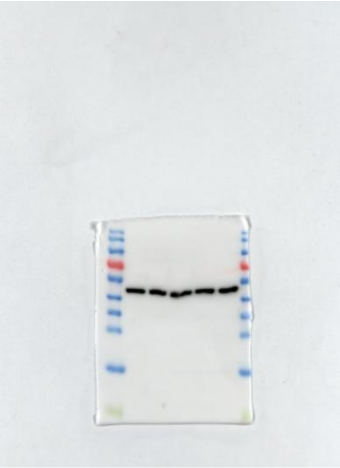

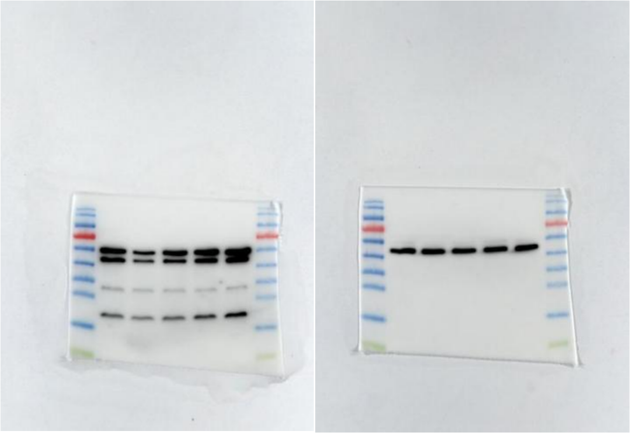

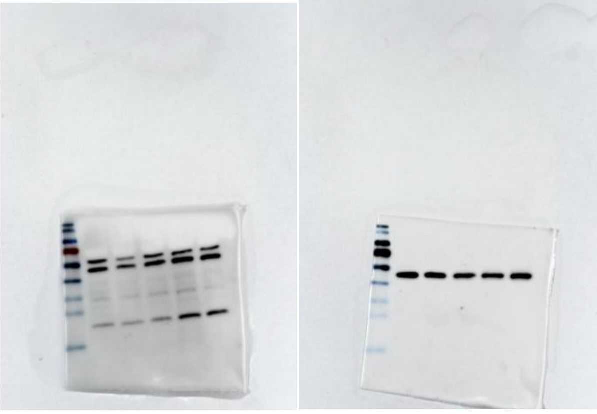

Figure 10L(The image on the left is GLUT1, and the corresponding housekeeping Protein is on the right):

GLUT1: **β-actin：**

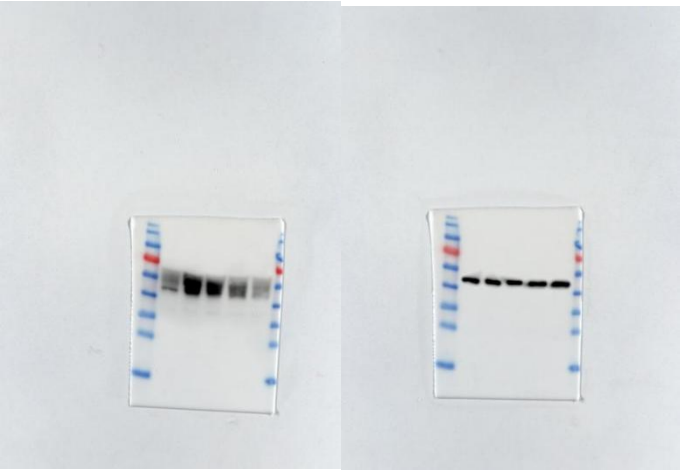

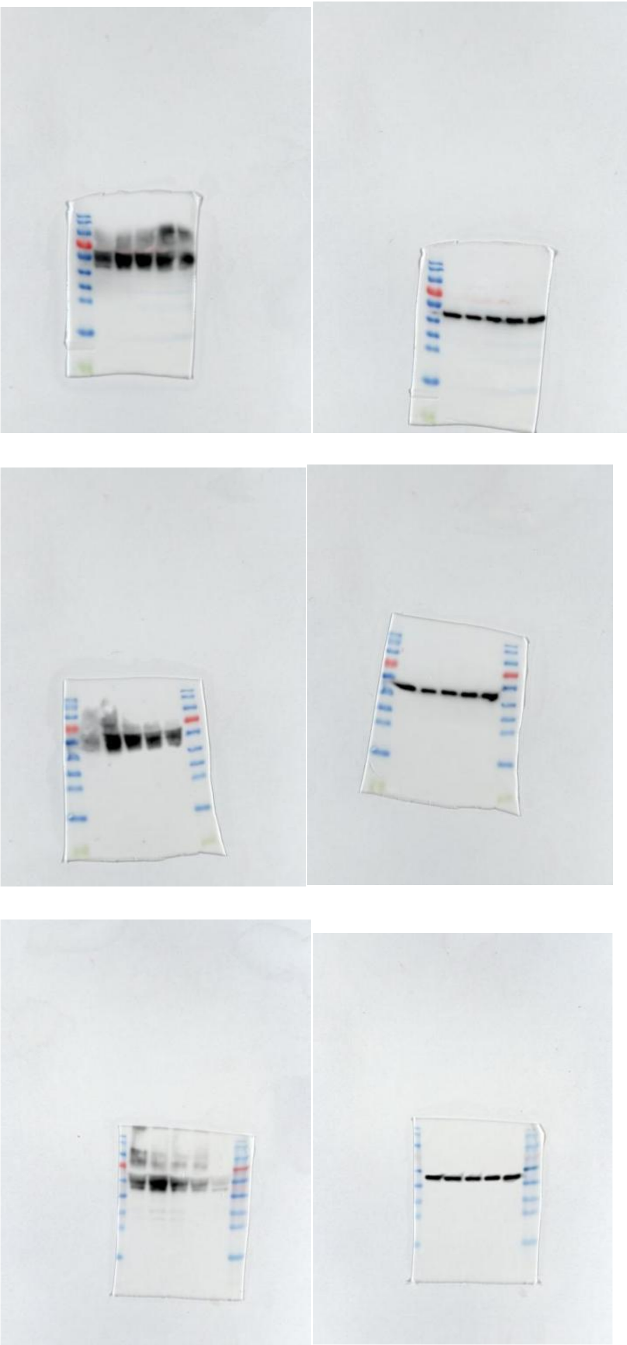

Figure 10L(The image on the left is LDH, and the corresponding housekeeping Protein is on the right):

LDH： **β-actin：**

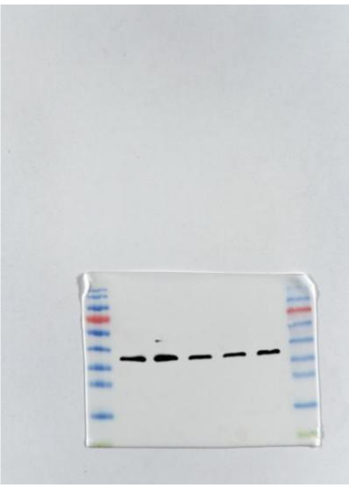
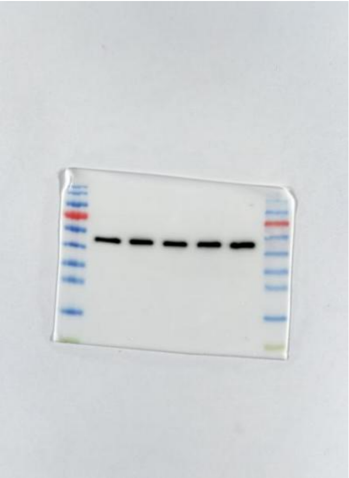
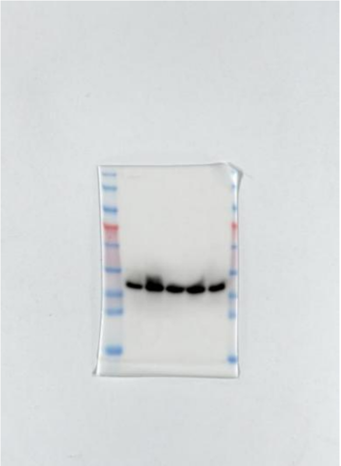
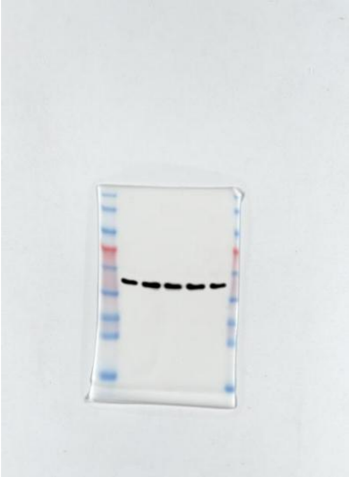
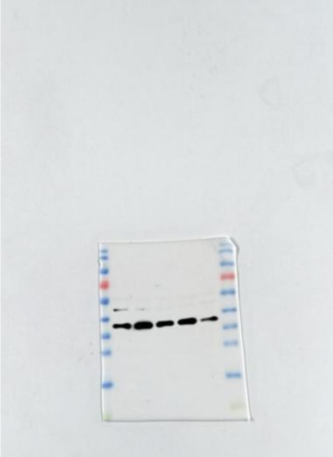
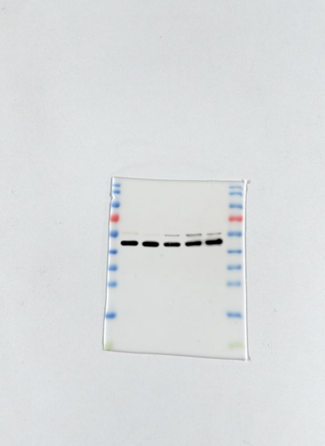

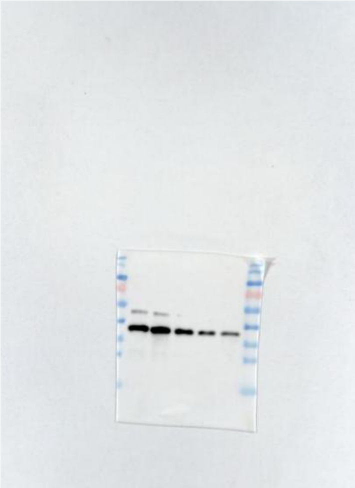
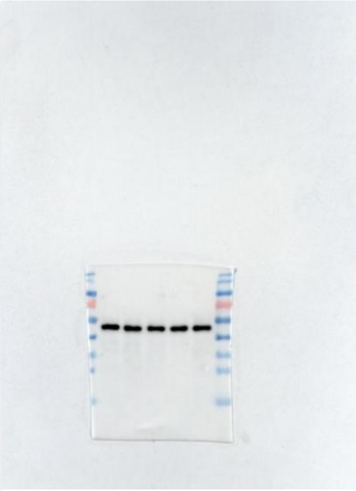

Supplement: Supplementary file 1 [file biomolecules-15-00742-s001.zip › Supplementary File.pdf]
